# Targeting the heparan sulfate proteoglycan/heparanase axis as a novel therapeutic strategy to drive adipogenesis and block tumor growth of dedifferentiated liposarcoma

**DOI:** 10.7150/ijbs.133802

**Published:** 2026-08-11

**Authors:** Cinzia Lanzi, Enrica Favini, Laura Dal Bo, Monica Tortoreto, Stefano Percio, Valentina Zuco, Gian Paolo Dagrada, Silvia Brich, Paola Collini, Loris De Cecco, Dario Callegaro, Roberta Sanfilippo, Alessandro Gronchi, Nadia Zaffaroni, Yehuda G. Assaraf, Sandro Pasquali, Giuliana Cassinelli

**Affiliations:** 1 Molecular Pharmacology, Department of Experimental Oncology, Fondazione IRCCS Istituto Nazionale dei Tumori, Milan, Italy; 2 Pathology Unit 2, Department of Advanced Diagnostics Fondazione IRCCS Istituto Nazionale dei Tumori, Milano, Italy; 3 Soft Tissue Tumor Pathology Unit, Department of Advanced Diagnostics, Fondazione IRCCS Istituto Nazionale dei Tumori, Milan, Italy; 4 Integrated Biology of Rare Tumors, Department of Experimental Oncology, Fondazione IRCCS Istituto Nazionale dei Tumori, Milan, Italy; 5 Department of Surgery, Fondazione IRCCS Istituto Nazionale dei Tumori, Milan, Italy; 6 Medical Oncology 2, Department of Cancer Medicine, Fondazione IRCCS Istituto Nazionale dei Tumori, Milan, Italy; 7 The Fred Wyszkowski Cancer Research Laboratory, Faculty of Biology, The Technion-Israel Institute of Technology, Haifa 3200003, Israel

**Keywords:** dedifferentiated liposarcoma, CX-01, syndecan-1, adipogenesis, c-Met, AKT

## Abstract

Dedifferentiated liposarcoma (DDLPS) is an aggressive mesenchymal malignancy coexisting with a low grade well-differentiated component. Pathways implicated in liposarcoma growth and dedifferentiation are promoted by heparan sulfate (HS) proteoglycans (HSPG) and their modifying enzymes including heparanase. HSPGs serve as co-receptors enhancing tyrosine kinase signaling and tumor aggressiveness. Targeting these interactions bears promise in attenuating liposarcoma growth. We employed an investigational HS mimetic, the non-anticoagulant heparin CX-01 (dociparstat), to assess its HS competition impact on deregulated adipogenic differentiation and growth of human DDLPS cell lines and patient-derived xenografts (PDXs). Remarkably, CX-01 reduced colony formation and invasive capacities of DDLPS cell lines, inducing cytoskeleton remodeling, lipid accumulation and reactivation of adipogenic program. Mechanistic studies into the anti-DDLPS activity of CX-01 unveiled Syndecan 1 (SDC1)/heparanase system and receptor tyrosine kinase-AKT signaling as targets of cell growth inhibition and induction of differentiation. CX-01 treatment of mice harboring DDLPS PDXs attenuated tumor growth, enhanced lipid content and consistently altered the transcriptome, modulating pathways associated with tumor dedifferentiation (adipogenesis and fatty acid metabolism) and tumor-microenvironment interaction (TGFβ signaling, inflammatory response). In two independent cohorts of DDLPS patients, genes downregulated in CX-01-treated PDXs (SDC1, TIMP1, FN1, COL5A1, and MMP14), were found preeminently expressed in the dedifferentiated, compared to the well-differentiated tumor component and normal fat. This suggests a role for these genes in disease progression. Collectively, this study demonstrates the remarkable potential of HS competition to simultaneously block multiple anti-adipogenic players representing metabolic vulnerabilities, and to promote a differentiated tumor phenotype markedly less aggressive.

## Introduction

Well differentiated liposarcoma (WDLPS) and dedifferentiated liposarcoma (DDLPS) are the most common adult-type malignant lipogenic tumors 1. WDLPS, characterized by the presence of tumor adipocytes of varying sizes, is a low-grade, slowly growing, locally aggressive disease. DDLPS is typically a non-lipogenic, grade 2 or 3 sarcoma with an intrinsic propensity for local recurrence and metastasis 1. In most cases, DDLPS is associated with a WD component. WDLPS and DDLPS exhibit giant chromosomes and supernumerary rings containing highly amplified sequences from the 12q13-15 chromosomal region 1, 2. Genomic amplifications of proto-oncogenes located in this region, particularly the mouse double minute 2 (*MDM2*) and cyclin-dependent kinase 4 (*CDK4*), occur in nearly 100% of cases. These drive liposarcomagenesis via negative regulation of the tumor suppressor p53, promotion of cell cycle progression and perturbation of the adipogenic differentiation program 2. Other recurrent genomic co-amplifications, prevalent in DDLPS, include receptor tyrosine kinase (RTK) signaling components like the fibroblast growth factor receptor substrate 2 (*FRS2*) residing in 12q15 2-4. Additional alterations contribute to the dedifferentiated phenotype and the aggressive behavior by impairing the function/expression of key mediators of adipocytic differentiation including the regulator peroxisome proliferator-activated receptor γ (PPAR-γ) 2. Moreover, distinct tumor microenvironment (TME) features are emerging as critical factors promoting disease progression 4-8.

While surgery is the primary treatment for localized liposarcoma, anthracycline-based regimen represents the standard first-line therapy for advanced disease, albeit with limited activity and severe side effects 2, 9. Clinical trials with targeted inhibitors of MDM2, CDK4 and RTKs failed to show a superior activity over standard therapy in metastatic patients 2, 10. Exploiting intrinsic tumor cell plasticity to promote differentiation represents an attractive approach in sarcomas presenting a bi-phenotypic morphology such as DDLPS 11. Elucidation of the complex regulatory networks driving liposarcoma dedifferentiation has the potential to uncover druggable vulnerabilities which can lead to the development of novel therapeutic modalities to enhance patients' outcome.

Heparan sulfate proteoglycans (HSPGs), the main components of cell surface and extracellular matrix (ECM), operate in concert with their modifying enzymes (e.g., heparanase), exerting a crucial role in development and differentiation pathways including adipogenesis. The HSPG system participates in the control of lipid homeostasis and adipocytic differentiation via the functional modulation of many heparan sulfate (HS)-binding biomolecules (e.g., growth factors/cytokines and their receptors, lipoproteins, lipases) and the regulation of gene expression 12. Cumulative evidence suggests a role for deregulation of components of this system in liposarcoma pathobiology 13-15. Specifically, increased expression levels of the HSPG syndecan 1 (*SDC1,* aka *CD138*) in DDLPS compared with WDLPS and normal adipose tissue, likely promoted by fibroblast growth factor (FGF)-mediated signaling, participates in the maintenance of proliferative potential as well as the dedifferentiated phenotype of the tumor 13. By virtue of their HS mimetic nature, heparin derivatives competitively interfere with the function of a vast array of HS-binding proteins including the endo β-D-glycosidase heparanase, growth factors, cytokines, adhesion molecules, inflammatory mediators and tissue-degrading enzymes with pivotal roles in cancer growth and progression 16. We previously demonstrated the ability of HS mimetics/heparanase inhibitors, in particular non-anticoagulant heparin derivatives, to counteract the growth and progression of different sarcoma types by interfering with HS-mediated processes such as TME cooperation, RTK signaling, and transcriptional/epigenetic regulation 15, 17-21. Based on the peculiar multi-factorial mechanism of action of heparin derivatives, we hypothesized that they may interfere with DDLPS aberrant differentiation and growth. Herein we explored the therapeutic potential of HS mimetics/heparanase inhibitors in DDLPS models. We assessed the anti-DDLPS activity *in vitro* and *in vivo* of the investigational CX-01 (ODSH, DSTAT, dociparstat), a 2,3-O-desulfated heparin which entered clinical trials 22-24, revealing its remarkable impact on tumor differentiation and growth.

## Materials and methods

### DDLPS PDXs and cell lines

DDLPS patient derived xenografts (PDXs) and cell lines were generated from patients with primary untreated, *MDM2* amplified, DDLPS of the retroperitoneum who underwent surgery at Fondazione IRCCS Istituto Nazionale dei Tumori, Milan (IT). The DDLPS LS-BZ-1 and LS-GD-1 cell lines were previously described 25. The newly generated LS-MMC-1 cell line was established from a surgical specimen from a patient with DDLPS of the retroperitoneum who underwent multivisceral resection and remained disease-free six years after surgery. The cell lines were cultured in DMEM F-12 medium (Lonza, Treviglio, Italy), supplemented with 10% fetal bovine serum (Euroclone, Pero, Italy). Analysis of cell STR Profile was performed by the Authentication Service of American Type Culture Collection (ATCC) (Manassas, VA). Cells were periodically tested for Mycoplasma with Mycoalert Mycoplasma Detection Kit (Lonza, Basel, Switzerland). Detailed description of LS-BZ-1 PDX generation and characterization was previously reported 25. The same procedure was used to establish the LS-MP-1 PDX from the tumor dedifferentiated component of a patient with retroperitoneal DDLPS. This patient underwent a multivisceral resection and developed disease recurrence 49 months after surgery. The consistency of LS-MMC-1 cell line and LS-MP-1 PDX with the originating clinical tumors was assessed by comparing morphology, *MDM2* amplification and transcriptomic profile following procedures described below and in Supplementary Information (SI).

### Drugs and *in vitro* cell treatments

CX-01 (ODSH, DSTAT, dociparstat) was provided by Cantex Pharmaceuticals (Weston, FL) and Chimerix Inc. (Durhan, NC). Detailed structural characteristics of CX-01 were previously reported 22, 26, 27. Basically, the removal of 2-O- and 3-O-sulfate groups reduced the heparin anticoagulant activity, enhanced heparanase inhibitory activity and improved safety margin without substantially affecting the anti-inflammatory properties 22.

For prolonged drug treatment schedules, cells were exposed to CX-01 dissolved in PBS for 72h, then the medium was replaced, and treatment repeated. The super-sulfated low molecular weight heparin ssLMWH, an oversulfated molecule characterized by average molecular weight of 6300 Da and reduced anticoagulant activity 16, 28, and the non-anticoagulant N-desulfated, 100% N-acetylated and 25% glycol split heparin SST0001 (roneparstat) 29 were included in the study for mechanistic validation. ssLMWH and SST0001were dissolved in sterile water.

Suppliers' details for SU11274, perifosine, OGT2115, and reagents are listed in Supplementary Table s1. SU11274 and OGT2115 were dissolved in dimethyl sulfoxide (DMSO) and further diluted in cell culture medium (final concentration of 0.2-0.25% DMSO). Perifosine was dissolved in sterile water. SU11274 was used in a range of drug concentrations reported as active in DDLPS cells 30.

### *In vitro* assays

Procedures for anchorage-independent growth, Matrigel invasion and branching morphogenesis assays, as well as detection of lipid content by BODIPY 493/503 and Oil red O staining, are described in detail in SI.

### Biochemical analyses, Western blotting, FISH, immunofluorescence microscopy and flow cytometry

Procedures and reagents used for proteomic profiling of phosphorylated RTKs, Western blot analysis, FISH, immunofluorescence microscopy and flow cytometry analyses are reported in SI and Supplementary Table s1.

### RNA interference

For *SDC1* silencing, 24h after plating, cells were transfected with specific or non-targeting siRNAs (20nM) (Supplementary Table s1) using RNAiMAX Lipofectamine (Thermo Fisher Scientific, Rockford, IL) in serum-free Opti-MEM I medium (Invitrogen, Carlsbad, CA). Cells were incubated with the siRNAs for 6h before addition of serum then processed for mRNA extraction, cell counting and flow cytometry analyses 72h later.

### RNA extraction and quantitative RT-PCR (qRT-PCR)

Extraction of total RNA from cells or frozen PDXs was performed by using RNeasy Plus Mini Kit (Qiagen, Hilden, Germany) and spectrophoto metrically analyzed using NanoDrop 2000c (Thermo Fisher Scientific, Waltham, MA) to assess purity and concentration. RNA was reverse transcribed using High-Capacity cDNA Reverse Transcription Kit (Applied Biosystems, Foster City, CA). cDNA amplifications were performed using TaqMan Universal Master Mix (Applied Biosystems) and the specific qPCR assays for *SDC1, HPSE* and *GAPDH* (Supplementary Table s1) in a 7900HT Fast Real-Time PCR System (Applied Biosystems). The 2^-ΔΔC^_T_ method was applied for analysis of relative gene expression data. *GAPDH* was chosen as an internal reference gene as its amplification efficiency, relative to the *SDC1* target gene, was stable with cDNA dilutions and its expression was not altered by *SDC1* silencing.

Information about the protocol for RNA extraction from clinical specimen was previously reported 31. Briefly, RNA was extracted with miRNeasy FFPE Kit (Qiagen) from FFPE material, quality-checked and quantified using the TapeStation 4150 (Agilent, Santa Clara, CA) and Qubit (Thermo Fisher Scientific), respectively.

### RNA-Seq data processing and analysis

We conducted quality control on the sequenced raw data using FastQC 32. Sequences were aligned to the human reference genome hg38 with STAR 33. Read counts were quantified based on the specific library preparation protocol employed. Raw counts were filtered by discarding reads that fell below the 10^th^ percentile of expression variance across samples or lacking an official gene symbol. Reads mapping to the same gene symbol were then collapsed by summing their counts. Transcriptomic matrix underwent normalization using the trimmed mean of M-value method, as implemented in the edgeR package 34 within the R environment. Both the gene expression data and the complete preprocessing pipeline have been deposited in the Gene Expression Omnibus (GEO) under accession number GSE307802. To assess the relationship between transcriptomic profiles of preclinical models (i.e., PDX and cell lines) and clinical tumor samples, we used a linear model and Spearman's coefficient to identify potential non-linear associations. In the same database other normalized datasets of patients were considered with accession numbers GSE159659 25, GSE296413 31 and GSE221492 5.

For all considered datasets, differential expression analysis was performed after addressing data heteroscedasticity with the voom method, also from the edgeR package 34. We employed a linear model from the limma package 35 to identify differentially expressed genes, quantifying both the log_2_ fold change (FC) and the t-statistic. To account for multiple comparisons and determine statistical significance, a false discovery rate (FDR) *p*-value correction threshold of 0.05 was applied. Gene set enrichment analysis (GSEA) was carried out using both the Hallmark collection from the Molecular Signature Database (MSigDB) and custom gene signatures. These custom signatures included those from existing literature 36 and selected gene sets retrieved from MSigDB using "SYNDECAN" as a query. Genes were ranked according to their t-statistic, a process facilitated by the fgsea package 37. A 0.05 FDR threshold was set to identify significantly enriched gene sets.

### *In vivo* experiments

Experiments were carried out using 6-week-old female CD1 Nude mice (NU/NUD CD1, RRID IMSR_CRL:086) for the PDX LS-BZ-1 or SCID mice (CB17/Icr-*Prkdcscid*/IcrIcoCrl, RRID: IMSR_CRL:236) for the PDX LS-MP-1 (Charles River, Calco, Italy) according to the strain of the donor mice. Animals were randomized into groups of 5-7 mice. Tumor fragments (about 3x3x3 mm) from donor mice were injected s.c. into the right flank of animals. Treatments started one day (LS-BZ-1) or 19 days (LS-MP-1) after tumor transplantation according to the growth rate of each of the two PDXs. CX-01 was dissolved daily in saline and administered s.c. twice daily at a dose of 30 mg/kg/injection, for 5 consecutive days per week, with treatments of 18 days for the faster growing LS-BZ-1 or 32 days for LS-MP-1 PDXs. CX-01 dose and scheduling were established based on previous *in vivo* studies using this drug and other heparin derivatives 17, 20, 38. Control mice received the drug vehicle. For drug efficacy assessment, tumor growth was monitored by biweekly measurements of tumor diameters with a Vernier caliper. Tumor volume (TV) was calculated according to the formula: TV (mm^3^) = d^2^ x D/2, where d and D, are the shortest and longest diameters, respectively.

### Statistical analysis

The TumGrowth model (https://kroemerlab. shinyapps.io/TumGrowth) was employed to examine the impact of treatment and time on tumor growth, also including random effects to account for inter-animal variability. The Mann-Whitney U-test was applied to compare two data distributions and the Jonckheere-Terpstra test was used to assess a monotonic behavior in an ordered set of categories using the GraphPad Prism software (version 4.0) (Graph- Pad Prism Inc., San Diego, CA). For *in vitro* experiments, the two-tailed Student's t test was used to compare two sets of data. *p* ≤ 0.05 was considered statistically significant.

## Results

### CX-01 affects DDLPS cell malignant features

**Figure 1:**
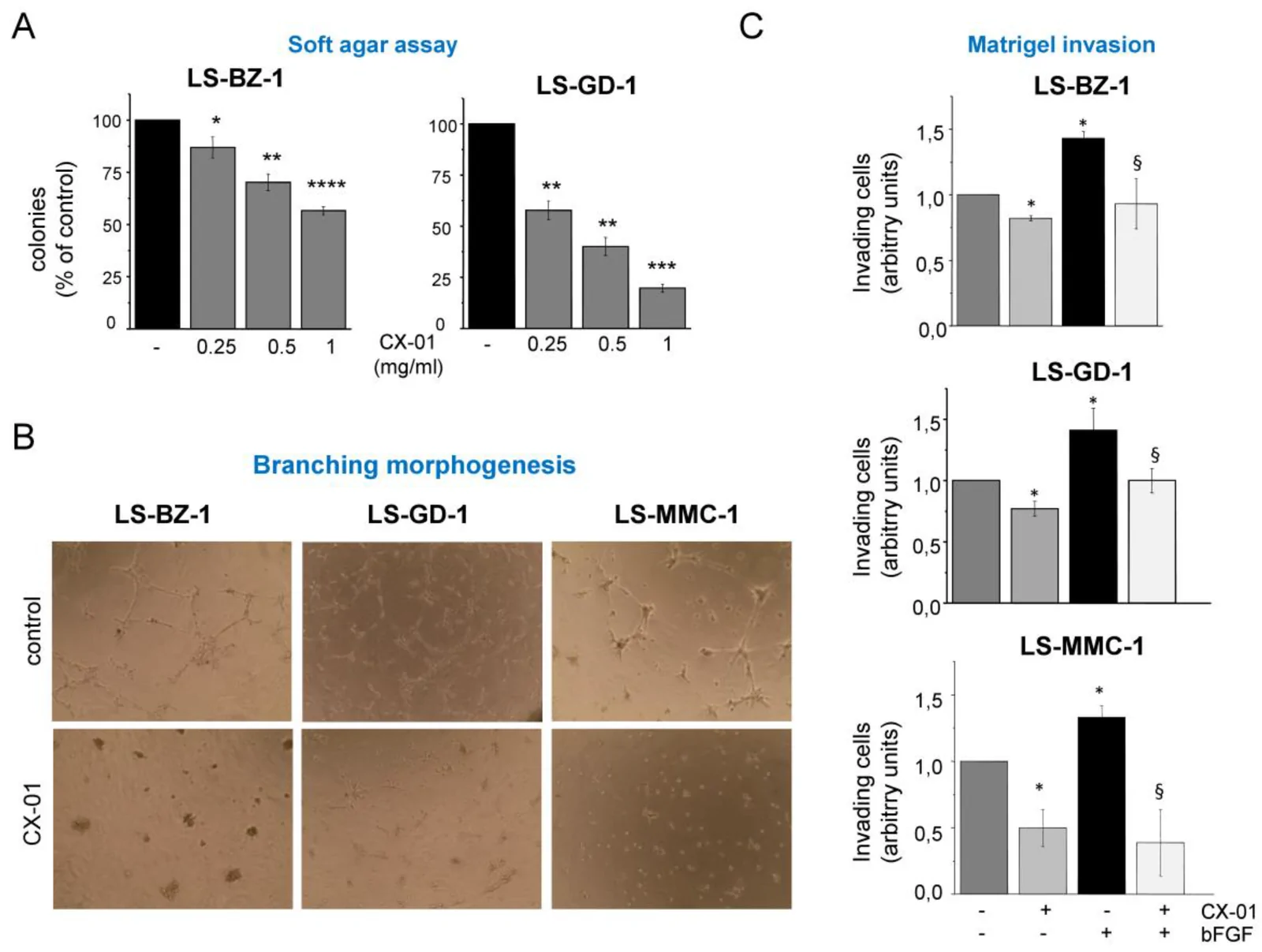
CX-01 treatment-induced inhibition of DDLPS cell anchorage-independent growth, morphogenic- and invasive-related abilities. A) Cells were seeded in soft agar in the presence or absence of CX-01 at the indicated concentrations. After 8-14 days, colonies were stained with a vital dye and counted under a magnifying projector. Data are reported as mean percentage of controls ± SD from three independent experiments performed in duplicate. *, *p* < 0.05, **, *p*< 0.01, ***, *p* < 0.001, ****, *p* < 0.0001 *vs* untreated cells. B) Control and drug-treated (1 mg/ml) cells were seeded in Matrigel-coated wells in serum-free medium for branching morphogenesis assay. Photos of cell networks were taken after 8h of incubation (original magnification 10x). C) Cells, pretreated with 1 mg/ml CX-01 for 24h, were transferred onto Matrigel-coated Transwell chambers and incubated for an additional 24h with (+) or without (-) bFGF (50 ng/ml). Invading cells are reported as arbitrary units ± SD from three independent experiments. *, *p* < 0.05 *vs* serum starved control; ^§^, *p* < 0.05 versus bFGF-stimulated cells.

The impact of CX-01 on the malignant phenotype of DDLPS cells was assessed using a panel of in-house generated tumor cell lines, obtained from untreated primary retroperitoneal DDLPSs. These included the previously reported LS-BZ-1 and LS-GD-1 cell lines 25 and the newly established LS-MMC-1 cell line. The latter exhibited typical WD/DDLPS cytogenetic alterations and *MDM2* amplification along with FRS2 overexpression. It also displayed a transcriptome profile consistent with the original clinical tumor (Supplementary Figures s1A-C). Treatment with CX-01 significantly inhibited the anchorage independent growth of LS-BZ-1 and LS-GD-1 cells in a dose-dependent manner (Fig. 1A). This assay was not applied to LS-MMC-1 cells which did not form colonies on soft agar. CX-01 also affected morphogenesis- and invasion-related abilities of the three tumor cell lines. In fact, as shown in Fig. 1B, the drug greatly reduced the formation of cellular networks in Matrigel, evidencing the ability of the drug to interfere with mechanisms underpinning directional migration, phenotypic rearrangements and intercellular organization in an artificial ECM. Moreover, this heparin derivative counteracted the invasive stimulus instigated by basic FGF (Fig. 1C), a prototypical growth factor that uses HSPGs as co-accessory molecules for effective receptor activation 39. This observation supports the ability of CX-01 to interfere with FGFR signaling, which is frequently upregulated in DDLPS 10.

### CX-01 promotes adipogenic differentiation in DDLPS cells

As heparin has been shown to stimulate adipogenesis 40, we examined the potential of the non-anticoagulant CX-01 to promote lipid accumulation in DDLPS cells.

**Figure 2:**
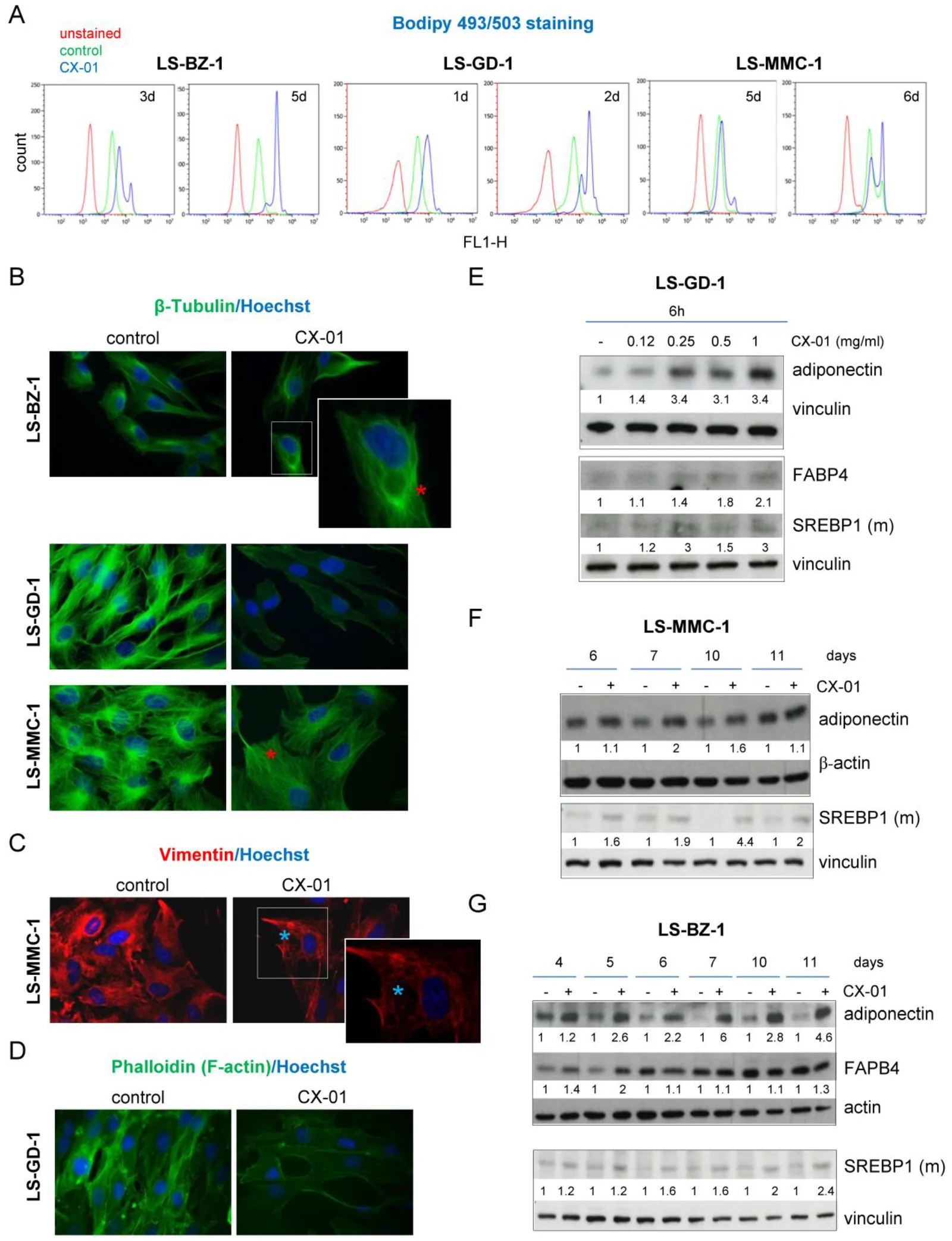
Lipid content enhancement, cytoskeleton reorganization and reactivation of adipogenic program induced by CX-01 treatment in DDLPS cells. A) Cells, exposed to 1 mg/ml CX-01 for the indicated days (d), were collected and incubated with Bodipy 493/503 for lipid content quantification by flow cytometry. Flow cytometry histograms show drug-induced accumulation of neutral lipids over time. B-D) Indirect immunofluorescence showing reorganization of cytoskeleton structures of β-tubulin (green), vimentin (red) and F-actin (green, phalloidin) in control and CX-01-treated cells (1 mg/ml for 72 h). Insets: enlarged details. Asterisks indicate cage-like structures deputed to lipid droplet accommodation. Nuclei are evidenced by Hoechst 33342 counterstaining (blue). Representative images are shown (original magnification 60x). E) Dose-dependent upregulation of the adipogenesis markers adiponectin, FABP4 and the mature form (m) of SREBP1 in LS-GD-1 cells following 6h of exposure to the indicated drug concentrations. F) and G) Upregulation of adiponectin, FABP4 and SREBP1 (m) in LS-BZ-1 and LS-MMC-1 cells over time. Cells were first treated with 1 mg/ml CX-01 for 72h, the medium was then replaced and CX-01 treatment repeated. Western blot images from one representative experiment are shown. Blots with antibodies recognizing actin isoforms and vinculin serve as loading controls. Numbers indicate the band intensity normalized to the respective loading control.

Flow cytometric analysis of cells stained with the fluorescent lipid dye BODIPY 493/503 evidenced a progressive increase in neutral lipid content in DDLPS cells treated with CX-01 (Fig 2A); this effect was detected earlier in LS-GD-1 cells. Oil Red O (ORO) staining confirmed the enhancement of lipid content in DDLPS cells upon CX-01 treatment (Supplementary Fig. s2).

A remarkable reorganization of the cytoskeleton is known to accompany the cellular shaping and structural adaptation necessary to accommodate lipid droplets during adipogenesis 41. Indeed, fluorescence microscopy analysis of cytoskeletal components (i.e. β-tubulin, vimentin and actin) in the DDLPS cell lines revealed the disruption of the microtubule network and organizing center, along with the occurrence of cage-like structures deputed to accommodate lipid droplets in drug-treated cells (Fig. 2B-2D and Supplementary Fig. s1D). Filamentous actin movement towards the cell periphery and reduction of stress fibers could also be noted in tumor cells exposed to CX-01 (Fig 2D and Supplementary Fig. s1D). This indicates that CX-01 treatment induced alterations in the DDLPS cell architecture, consistent with reduced anchorage-independent growth and adipocytic differentiation.

In accord with a reactivation of the adipogenic program, upregulation of adipogenesis-related molecules, such as the adipokine adiponectin, the transcriptional factor sterol regulatory element binding-protein-1 (SREBP1) and the fatty acid-binding protein 4 (FABP4) 11, 41, was observed in DDLPS cells upon CX-01 treatment (Fig. 2E-2G). To assess whether the ability to inhibit DDLPS anchorage-independent growth and stimulate adipocytic differentiation was also shared by other heparin derivatives, we examined the impact of the highly reactive super-sulfated low molecular weight heparin (ssLMWH), a preclinical molecule endowed with reduced anticoagulant activity 20, 28. ssLMWH effectively blocked DDLPS colony formation, induced adipogenesis markers and lipid accumulation (Supplementay Fig s3A-3C)*.* These findings support the potential of the heparin class of molecules to exploit the yet proficient tumor cell molecular machinery to drive DDLPS cells towards a more differentiated adipocytic phenotype.

### Inhibition of RTK-AKT pathways contributes to CX-01-induced adipogenic differentiation

**Figure 3:**
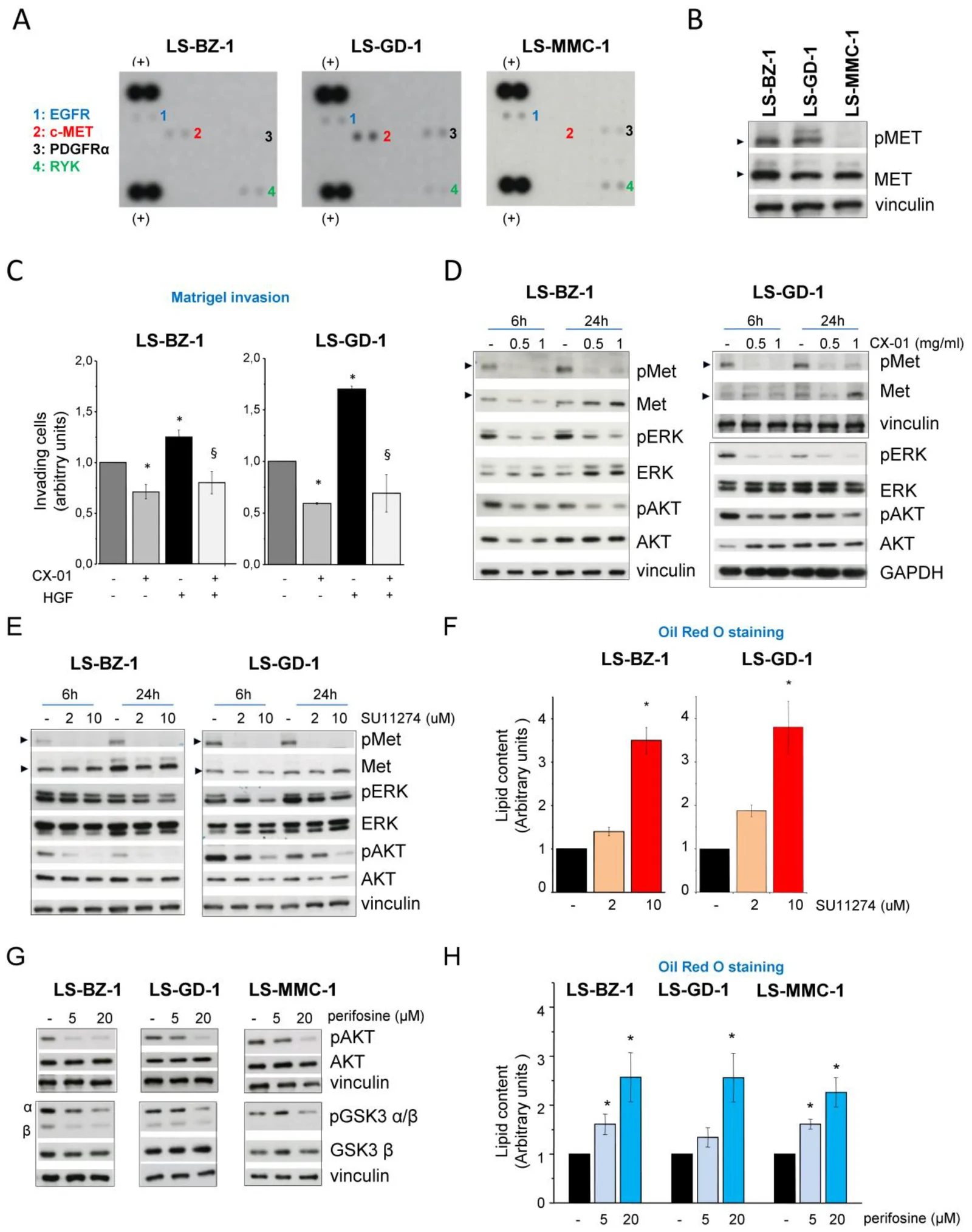
Inhibition of c-Met and AKT activation in CX-01-treated DDLPS cells. A) Phospho-protein arrays revealing basal RTK activation in the three cell lines. (+), reference spots. B) Western blots showing levels of c-Met tyrosine phosphorylation and expression. Triangles indicate the mature receptor form. C) Matrigel invasion assay. Cells, pretreated with 1 mg/ml CX-01 for 24h, were transferred onto Matrigel-coated Transwell chambers and incubated for additional 24h with (+) or without (-) HGF (50 ng/ml). The number of invading cells is reported as arbitrary units ± SD from three independent experiments performed in duplicate. *, *p* < 0.05 *vs* serum starved controls; ^§^, *p* < 0.05 *vs* HGF-stimulated cells. D) Western blots showing inhibition of c-Met signaling activation (pAKT and pERK) upon CX-01 treatment at the indicated concentrations and exposure times. E) Western blots showing inhibition of c-Met signaling upon treatment with the c-Met selective small molecule inhibitor SU11274. F) Enhancement of lipid content in cells treated with SU11274 for 24h. Quantitative detection of neutral lipids was performed by Oil Red O staining and spectrophotometric analysis. G) Western blots showing inhibition of AKT signaling (pGSK3β) upon treatment with perifosine, an AKT small molecule inhibitor for 72h. H) Enhancement of lipid content detected by Oil Red O staining in cells treated with perifosine for 72h. In F) and H), OD readings were corrected for sample protein content and data reported as arbitrary units ± SE from at least three independent experiments. *, *p* < 0.05 drug-treated *vs* control cells. In B), D), E) and G), filters were first probed with phospho-specific antibodies, were then stripped and reprobed with antibodies directed against the respective proteins. Vinculin and GAPDH are loading controls. Images from one representative experiment are shown.

An aberrant activation of RTKs and downstream signaling has been implicated in DDLPS cell proliferation and deregulation of the adipogenic differentiation program 41, 42. Given the potential of HS mimetics like heparin derivatives to modulate RTK activation 39, we first applied an antibody array-based phospho-proteomic approach to assess the activation status of multiple RTKs in DDLPS cells (Fig. 3A). The three tumor cell lines showed different levels of activation of a few RTKs including EGFR, RYK, PDGFRα and c-Met. We focused our investigation on c-Met as the hepatocyte growth factor (HGF)/c-Met axis participates in key interactions between adipocytes and the surrounding microenvironment 12, 42. Moreover, c-Met has been implicated in DDLPS pathobiology and was proposed as a potential therapeutic target 10, 30, 43. Western blot (WB) analysis confirmed c-Met expression in the three cell lines and high tyrosine phosphorylation in LS-BZ-1 and LS-GD-1 cells (Fig 3B). Cell invasion was enhanced by HGF in a Matrigel invasion assay, indicating that c-Met maintained ligand responsiveness in all three cell lines despite constitutive activation in LS-BZ-1 and LS-GD-1 cells (Fig. 3B-3C and Supplementary Fig. s1E).

We previously showed that heparin derivatives can interfere with the activation of c-Met which uses HSPGs as accessory molecules in sarcoma cells 19. Treatment with CX-01 inhibited DDLPS cell invasiveness and counteracted the pro-invasive stimulus of HGF (Fig. 3C and Supplementary Fig. s1E). WB analysis corroborated the ability of CX-01 to interfere with the activation of c-Met and the downstream ERK and AKT pathways, which are implicated in the receptor cascade in DDLPS cells 30 (Fig. 3D). Similarly, inhibition of c-Met, AKT and ERK activation was observed in LS-BZ-1 and LS-GD-1 cells exposed to ssLMWH (Supplementary Fig. s3D).

To assess the role of c-Met and downstream signaling inhibition on lipid accumulation, ORO staining was performed in cells treated with SU11274, a highly selective inhibitor of the c-Met RTK which exerts its activity as an ATP competitor; this Met kinase inhibitor was previously shown to display anti-proliferative/migratory activity in DDLPS cells 30. Abrogation of c-Met phosphorylation and inhibition of downstream AKT and ERK activation induced by SU11274 (Fig. 3E) were associated with a significant enhancement of lipid droplets in LS-BZ-1 and LS-GD-1 cells (Fig. 3F and Supplementary Fig. s4A). Expectedly, ORO staining did not evidence a significant lipid content increase in LS-MMC-1 cells exposed to the c-Met inhibitor (data not shown).

The AKT pathway, which supports human mesenchymal stem cell mitotic clonal expansion and blocks adipogenic differentiation, has been also involved in the molecular pathogenesis of WDLPS and DDLPS with implications for tumor aggressiveness 44, 45. We thus used a selective AKT inhibitor to assess the contribution of this kinase to the regulation of lipid accumulation in DDLPS cells. Treatment with the alkyl-phospholipid perifosine, targeting the pleckstrin homology (PH) domain of AKT, hindered the kinase activation and downstream signaling as evidenced by the reduced phosphorylation of the GSK3α/β substrate (Fig. 3G). Interference with AKT signaling resulted in a significant enhancement of lipid content in all three DDLPS cell lines (Fig. 3H and Supplementary Fig. s4B). Considering that LS-MMC-1 cells lack c-Met overactivation, it is likely that overexpression and hyperactivation of FRS2 (Supplementary Fig. s1B) contribute to the constitutive activation of AKT signaling in these tumor cells 46. These findings reveal that AKT activation promoted by constitutive upstream signaling (e.g., c-Met in LS-BZ-1 and LS-GD-1 cells and presumably FRS2 in LS-MMC-1 cells), is a central node hindering DDLPS cell differentiation.

### Downregulation of multiple HS mimetic targets, promotes adipogenic differentiation and reduces the proliferative potential of DDLPS cells

**Figure 4:**
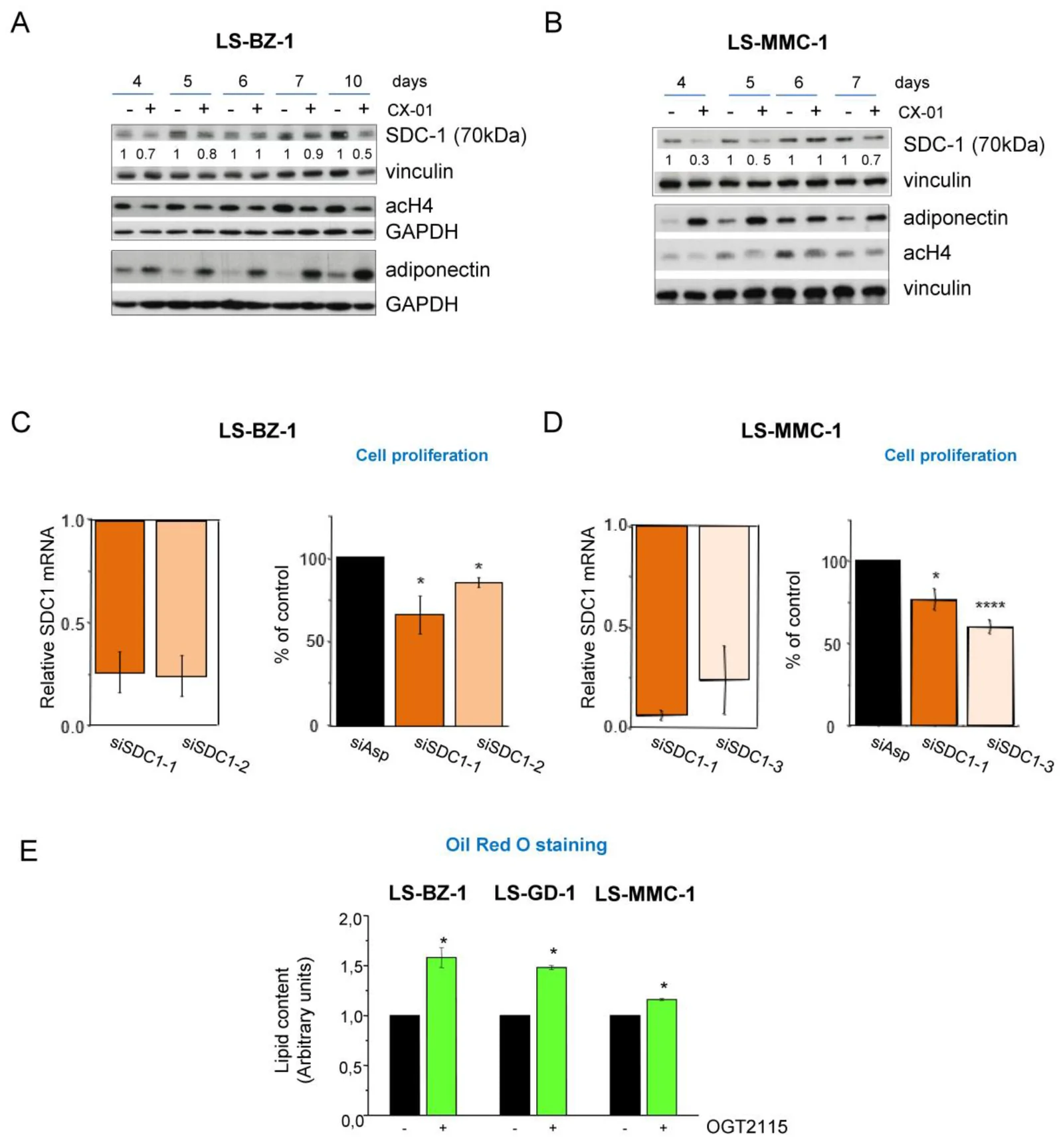
Downregulation of the HS mimetics' targets SDC1, heparanase and p300 histone acetyltransferase are associated with adipogenic differentiation and reduced proliferation of DDLPS cells. A) and B), CX-01-induced adipocytic differentiation is accompanied by downregulation of SDC1 and histone H4 acetylation. Time-course experiments show drug-induced reduction of SDC1 protein levels and acetylation of histone H4, a p300 target, in parallel with upregulation of adiponectin. LS-BZ-1 and LS-MMC-1 cells were exposed to 1 mg/ml CX-01 for 72h, then the medium was replaced and drug treatment repeated. Blots from one representative experiment are shown. Vinculin and GADPH represent actual protein loading. The intensity of the bands was normalized to the respective loading controls. C) and D) *SDC1* knockdown inhibits DDLPS cell proliferation. After a 72h-transfection with 20 nM non-specific RNA oligonucleotide (siAsp) or SDC1 siRNAs (siSDC1-1 and siSDC1-2 for LS-BZ-1 cells; siSDC1-1 and siSDC1-3 for LS-MMC-1 cells), cells were processed for qRT-PCR analysis of *SDC1* expression or, alternatively, were counted. Left panels, mean relative *SDC1* mRNA values ± SE referred to siAsp samples. Right panels, cell number reported as mean percentage of controls ± SE from at least three independent experiments. *, *p*<0.05; ****, *p*<0.0001 *vs* siAsp-transfected cells. E) Enhanced DDLPS cell lipid content induced by heparanase inhibitor OGT2115. Cells were exposed to solvent or 1 µM OGT2115 for 72h, then neutral lipid content was assayed by spectrophotometric measurement of Oil Red O staining. OD readings were corrected for sample cell number and data are reported as arbitrary units ± SE from three independent experiments. *, *p* < 0.05 drug- *vs* solvent-treated cells.

We next examined the involvement of three additional well-recognized HS mimetic targets, the HSPG SDC1, the p300 histone acetyltransferase (HAT) and the endo β-D-glycosidase heparanase 16, 22, 47, in the pro-differentiation and growth-inhibitory effects of CX-01. In CX-01-treated cells, a gradual increase in adiponectin was accompanied by downregulation of SDC1 at both protein and mRNA levels (Fig. 4A-4B and Supplementary Fig. s5A-5B-5C). Western blots evidenced that CX-01 induced a reduction of both the SDS-resistant 70 kDa dimer and the 30 kDa core protein (Fig. 4A-4B and Supplementary Fig. s5A) 48, a feature shared by other, structurally different, heparin derivatives such as ssLMWH and SST0001 (Supplementary Fig. s3E).

These effects are consistent with previously reported findings suggesting a role for SDC1 as a proliferation promoter and differentiation inhibitor in undifferentiated adipocytic progenitors 13. In fact, transient SDC1 knockdown by siRNAs resulted in a reduction of the proliferative potential of DDLPS cells (Fig. 4C-4D and Supplementary Fig. s5D). Heparins, including CX-01, have been reported to directly block the p300 HAT, a crucial histone acetyltransferase in epigenetic regulation of gene expression 47. Accordingly, a reduction in histone H4 acetylation was observed in CX-01-treated cells (Fig. 4A- 4B, Supplementary Fig. s5A).

CX-01 also shares with other HS mimetics the ability to inhibit heparanase 22, the only mammalian endo β-D-glycosidase that, by cleaving the HS chains of HSPGs into discrete active fragments, modulates the multiple HSPG functions affecting tumor cells and their interactions with the TME 15, 49. We used the small molecule OGT2115 to specifically block heparanase activity. Indeed, enhanced lipid accumulation was observed in all three treated DDLPS cell lines, indicating that heparanase-dependent HS degradation participates in the negative regulation of adipocytic differentiation (Fig. 4E). Analogously to other structurally different non-anticoagulant heparins and heparanase inhibitors such as ssLMWH and the glycol-split derivative SST0001 21, 50, CX-01 inhibited heparanase functions also by downregulating its expression likely through a transcriptional mechanism (Supplementary Fig. s3E and Supplementary Fig. s5A-5B).

Overall, these findings indicate that SDC1 and heparanase are both involved in the deregulation of DDLPS cell differentiation and proliferation and that their impacts can be counteracted by HS mimetics such as CX-01 which can promote adipogenesis-related processes likely influencing epigenetic regulation as well.

### CX-01 attenuates the growth and alters the transcriptomic profile of DDLPS PDX

**Figure 5:**
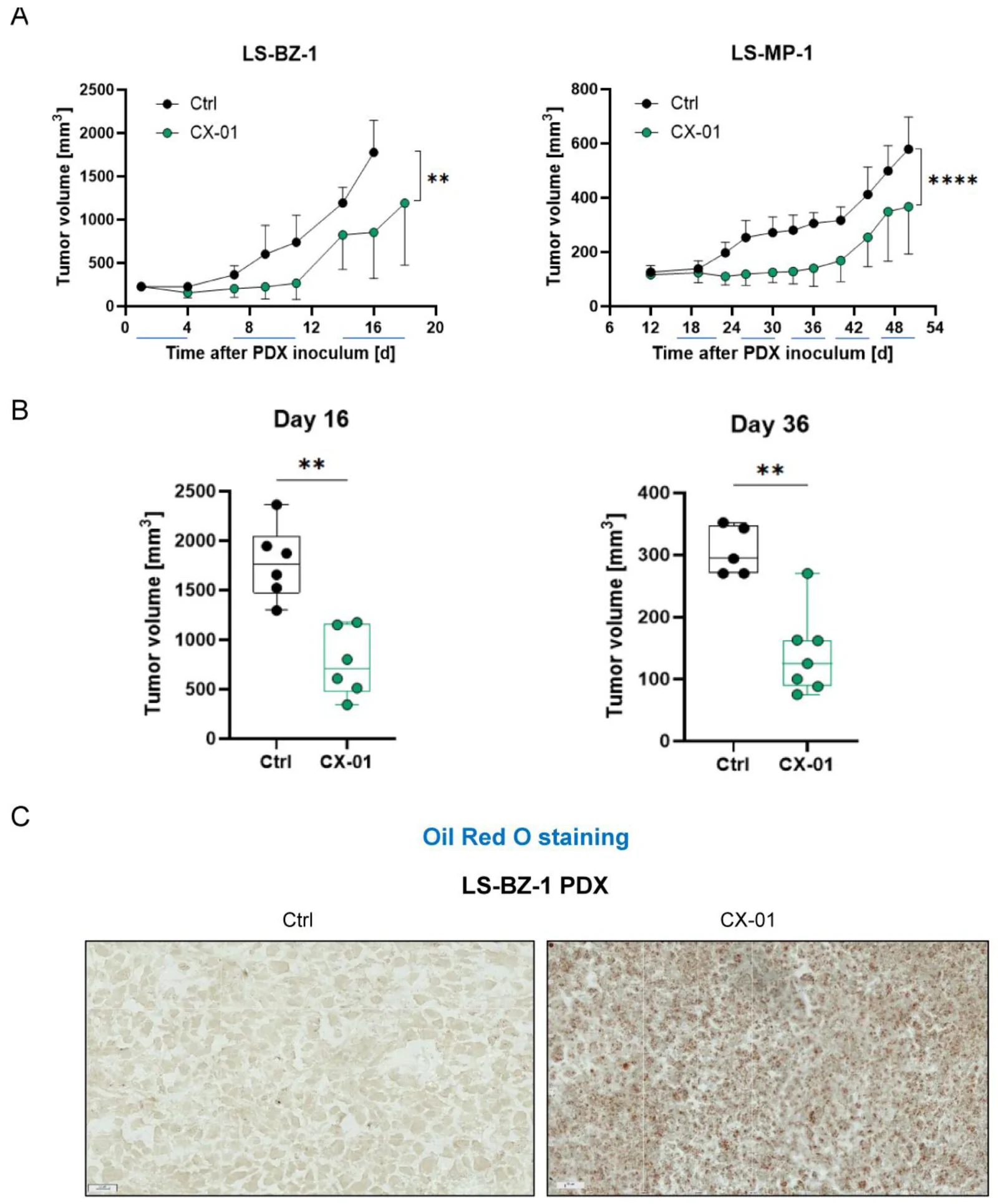
Treatment with the heparin derivative CX-01 inhibits growth and enhances lipid content of DDLPS PDX. A) Growth curves of LS-BZ-1 and LS-MP-1 PDXs. Animals were treated with vehicle (Ctrl) or CX-01 s.c. at 30 mg/kg, 2qdx5/w. LS-BZ-1 harboring nude mice were treated for 18 days starting 1 day after tumor transplantation. LS-MP-1 harboring SCID mice were treated for 32 days starting 19 days after tumor transplantation. Each point represents the mean tumor volume in 5/8 mice ± SD. The TumGrowth model was used to test the effect of treatment and time on tumor growth giving significance referred to the comparison of the entire curves. **p < 0.01 and ****p<0.0001. B) Tumor volume distribution (Min to Max box plots with all points showed) in experimental groups reported in A) at day 16 (LS-BZ-1) and 36 (LS-MP-1). **p < 0.01. C) Oil Red O staining performed on frozen PDX tissue slices showing increased content of lipid droplets in LS-BZ-1 tumors from mice receiving CX-01 with respect to untreated tumors. Representative images are shown (scale bar 20 µm).

To address whether the effects of CX-01 observed in DDLPS cell cultures were reflected in antitumor activity, we performed *in vivo* experiments using two DDLPS PDXs established from untreated primary retroperitoneal DDLPSs. As previously reported, the LS-BZ-1 PDX recapitulated the histological/genomic features of the clinical tumor and low sensitivity to doxorubicin 25. Similarly, the newly established LS-MP-1 PDX showed MDM2, CDK4, FRS2 overexpression, and a transcriptomic profile consistent with the original tumor (Supplementary Fig. s6A-6C). Treatment with CX-01 of LS-BZ-1-carrying mice significantly blocked tumor growth during the first two courses (10 days), achieving a maximum tumor volume inhibition (max TVI) of 64% (day 11, *p* < 0.05) which was higher than the max TVI (40%) attained by the gold standard doxorubicin in this model 25. Administration of CX-01 to LS-MP-1-carrying mice significantly inhibited tumor growth during the first three courses (18 days), achieving a max TVI of 54% (days 30-36) (Fig. 5A and 5B). Thereafter, treated tumors resumed growth during the pause treatment although maintaining a significant TVI up to day 16 (LS-BZ-1 52%, *p* < 0.01) and day 50 (LS-MP-1, 37%, *p* < 0.05). These *in vivo* drug treatments were well tolerated. The drug ability to promote adipogenic differentiation in *in vivo* setting was confirmed by ORO staining which showed a remarkable enhancement of neutral lipid droplets in PDX tissue samples from CX-01-treated mice compared to controls (Fig. 5C). In addition, western blotting of *ex vivo* PDX samples provided pharmacodynamic validation of CX-01-induced modulation of key players such as AKT, SDC1 and HAT (Supplementary Fig. s7A).

**Figure 6:**
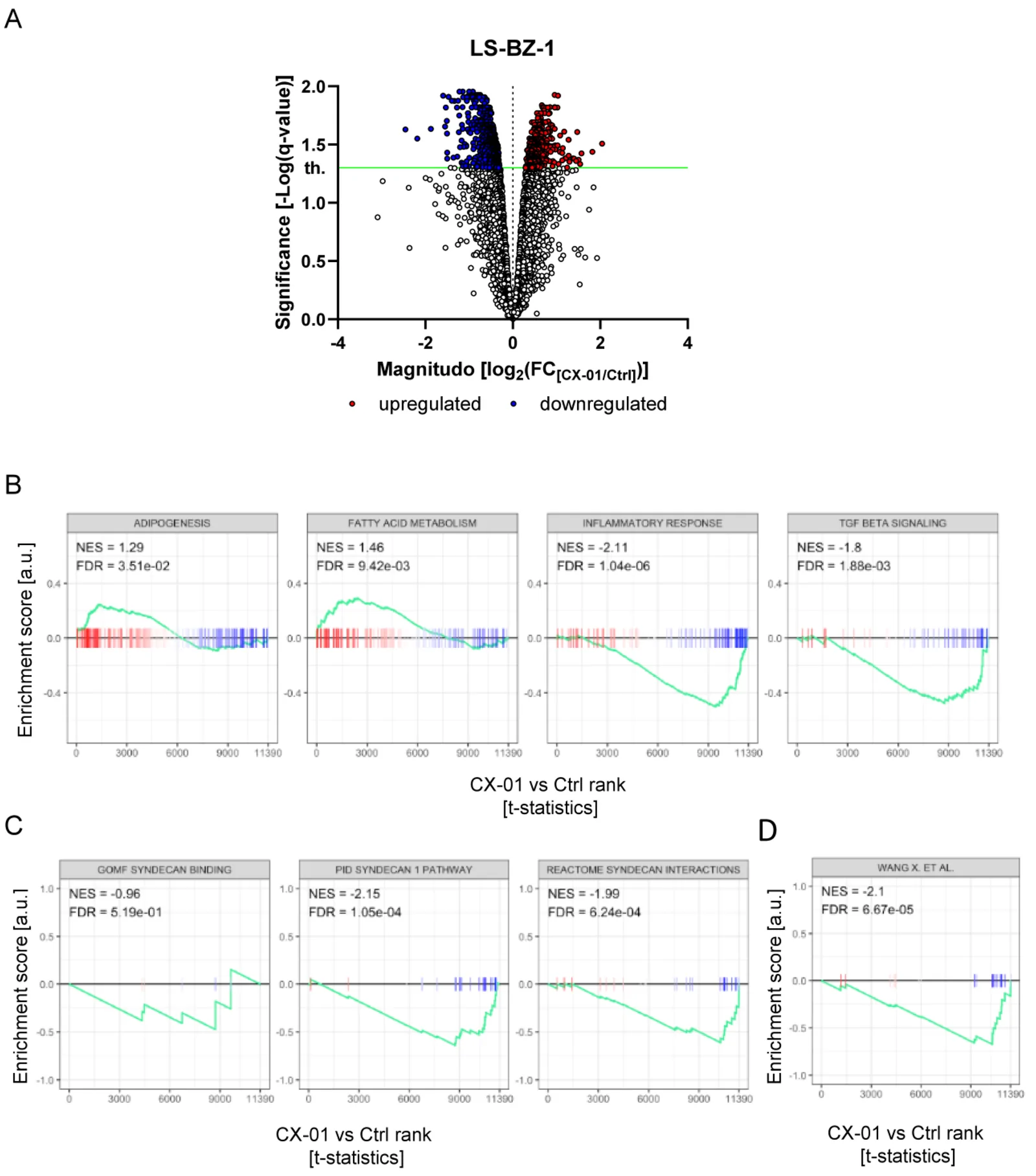
Gene set enrichment analysis (GSEA) of CX-01 treatment-induced perturbations in LS-BZ-1 PDX. A) Volcano plot of DEGs in CX-01-treated *vs* Ctrl LS-BZ-1 PDX. Out of 1271 statistically significant DEGs, 465 genes were upregulated (red) and 806 were downregulated (blue) upon drug treatment. B) Enrichment plots of selected Hallmark collection gene sets (Adipogenesis, Fatty acid metabolism, Inflammatory response, TGFβ signaling). C) Enrichment plots of syndecan-related gene sets of MSigDB (PID syndecan 1 pathway, REACTOME syndecan interactions, GOMF syndecan binding). D) Enrichment plots of the reported Wang *et al.* signature 36 distinguishing between DDLPS and WDLPS.

To gain further insights into the molecular mechanisms underlying the activity of CX-01 in DDLPS, we performed RNA-Seq on three untreated as well as treated LS-BZ-1 PDX samples collected at the end of treatments. The transcriptomic analysis revealed 1271 differentially expressed genes (DEGs) including 806 downregulated and 465 upregulated genes, considering logFC ≥ 0 and q-value < 0.05 (Fig. 6A). Gene set enrichment analysis (GSEA) identified significant down-regulation of “Inflammatory response” and “TGF beta signaling”, as well as up-regulation of “Adipogenesis” and “Fatty acid metabolism” Hallmark collections of MSigDB (Fig. 6B). DEGs also related to adipogenesis included *FGF2, TIMP1*,* MYLK, FN1* (downregulated) and *MYH3, TNFSF12, FABP5* (upregulated) (Supplementary Table s2). A marked downregulation of genes in the inflammatory response was consistent with the reported anti-inflammatory activity of CX-01 38, 47, 51. Notably, among genes in this enriched gene set, *CXCL8* is transcriptionally regulated by the CXCL12-CXCR4 axis, a known CX-01 target 51. The downregulation of genes related to transforming growth factor beta (TGFβ) signaling revealed an additional signaling pathway affected by the heparin derivative. Indeed, apart from the Hallmark leading edge genes, other DEGs can be ascribed to this signature, including *TGFBR2, LRRC32, MMP14, TIMP1* (Supplementary Table s2). Functional *in vitro* validation of CX-01 interference with TGFβ activation was achieved by inhibition of the phosphorylation of SMAD2, a TGFβ signaling effector, and downregulation of TIMP-1, a TGFβ signaling transcriptional target 52, in DDLPS cells undergoing adipogenic differentiation (Supplementary Fig. s7B-8E). Focusing on *SDC1*-related genes, we selected gene sets of MSigDB employing “syndecan” as query. As depicted in Fig. 6C, the results uncover consistently significant enrichment in all gene sets, except for one gene set including only a few genes, which, despite showing the same trend, did not attain statistical significance.

We further examined the expression of 29 genes, recently identified in an integrated lipidomic and transcriptomic analysis, presenting higher expression in DDLPS compared to WDLPS clinical specimens 36. We found a significant enrichment of these genes among DEGs negatively affected by CX-01 treatment (Fig. 6D). Notably, some DEGs in this list, i.e., *TIMP1* (Tissue inhibitor of metalloproteinase 1), *FN1* (Fibronectin 1), *COL5A1* (Collagen type V alpha 1 chain), and *MMP14* (Matrix metalloproteinase 14), were present in several functional gene sets affected by CX-01 treatment (Supplementary Table s2). CX-01-induced downmodulation of FN1 protein in LS-BZ-1 PDX is shown in Supplementary Fig. s7F.

### PDX treatment with CX-01 affects the expression of genes highly expressed in the “DD” component of clinical DDLPS

**Figure 7:**
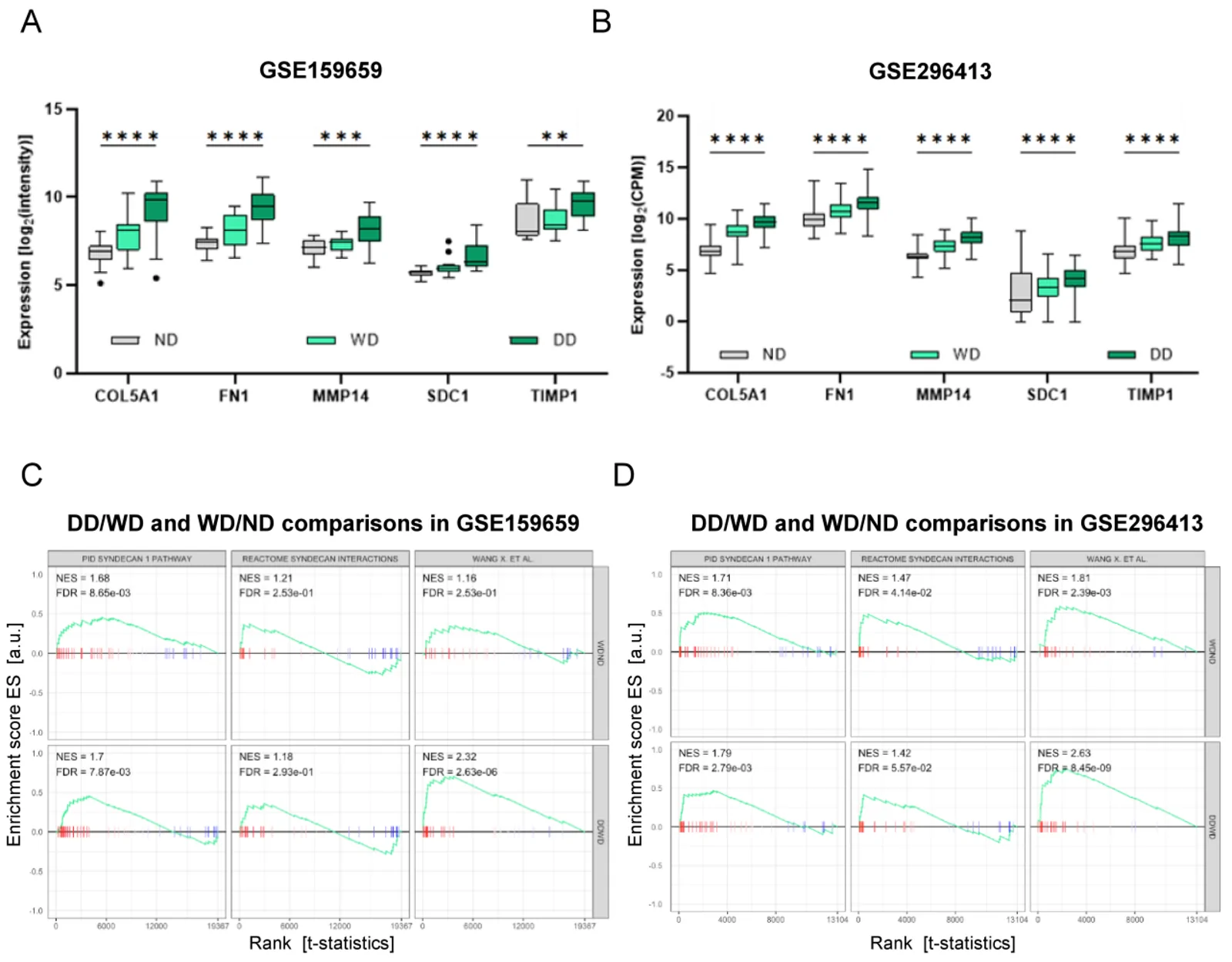
Transcriptomic analysis of RNA-Seq data from normal fat tissue (ND) and the WD or DD tumor components. Samples were obtained from the same sites of DDLPSs in two independent datasets, GSE159659 (n = 15, A, C) and GSE296413 (n = 68, B, D). A) and B), Boxplots show an increasing expression of selected genes (*COL5A1, FN1, MM14, SDC1* and* TIMP1*) from ND to the WD and DD tumor components. C) and D) GSEA enrichment plots show an enrichment towards upregulated genes in both the WD *vs* ND and DD *vs* WD comparisons for the syndecan-related gene sets (PID syndecan 1 pathway, REACTOME syndecan interaction) and the Wang *et al*., signature 36.

The transcriptomic analysis of LS-BZ-1 PDXs indicated that CX-01 treatment downregulated *SDC1* and other genes characterizing the DDLPS phenotype in association with a pro-differentiation effect. We therefore asked whether the expression of these genes reflected DDLPS progression/aggressiveness. We took advantage of available transcriptomic profiles of two series of patients with untreated primary retroperitoneal DDLPS to analyze gene expression in paired DD and WD tumor components, and normal adipose (ND) tissue obtained from the same resected specimens. In two series of primary DDLPS (GSE159659 and GSE296413) from patients who underwent surgery in our institute 25, 31, a significant monotonic increase in *COL5A1, FN1, MM14, TIMP1*, and *SDC1* levels was observed in the DD compared to the WD component, as well as in the WD component with respect to ND (Fig. 7A-7B). This finding was also corroborated in an independent external sarcoma series (GSE221492) 5 characterized by sample collection similar to the above cohorts with the sole exception of ND components (Supplementary Fig. s8).

We then interrogated the transcriptomes of DDLPSs in our institutional clinical series regarding gene sets which were downregulated upon CX-01 treatment in the DDLPS PDX (Fig. 6B). GSEA comparing WD *vs* ND as well as DD *vs* WD, showed a general trend towards an upregulation of either PID “syndecan 1 pathway” and REACTOME “syndecan interactions” gene sets as well as the Wang *et al*., signature 36, suggesting a plausible relevance in disease progression (Fig. 7C-7D).

Overall, these transcriptomic analyses in DDLPS models indicate that CX-01 downmodulated *SDC1*-related genes and additional genes which are upregulated in the DD component of the tumor. These findings support the potential of CX-01 to promote a transcriptional profile in DDLPS models associated with a more differentiated and less aggressive disease in liposarcoma patients.

## Discussion

WDLPS and DDLPS share main cytogenetic alterations and oncogene amplifications. Molecular mechanisms driving dedifferentiation have not been clearly elucidated and alterations beyond the known drivers present in DDLPS may enhance tumor cell proliferation and interfere with adipogenic program and lipid metabolism. A contribution of the TME interplay to DDLPS progression and dismal prognosis is also emerging 5, 6, 8, 36. Interfering with signaling pathways characteristic of DDLPS represents an attractive therapeutic approach; this is supported by recent reports showing that DDLPS cells retain biological features of their progenitors including pluripotency and adipocytic differentiation capacity 5, 45. Attempts to stimulate DDLPS adipocytic differentiation through the use of PPAR-γ agonists (e.g., pioglitazone) have produced disappointing results in the clinical setting 9 likely due to intrinsic PPARγ functional impairment and to the multifactorial nature of adipocytic differentiation deregulation 2, 4, 53.

In this study, we showed for the first time that a HS mimetic/heparin derivative reactivated the adipogenic program in DDLPS cells and exerted an anti-tumor activity in DDLPS PDXs. Cumulative experimental and clinical evidence implicates a deregulated HSPG/heparanase axis in the growth and progression of several sarcoma histotypes 14, 15. Targeting this pathway by non-anticoagulant heparins (e.g., roneparstat, ssLMWH) resulted in a significant inhibition of growth and metastatic dissemination of soft tissue and bone sarcoma models. It further resulted in an enhanced efficacy of both cytotoxic and targeted therapeutics, clinically relevant to these malignancies 18-21. The broad activity of heparin derivatives across different sarcomas is related to their HS mimetic nature which endows them with the ability to concomitantly affect multiple effectors acting in pathobiologically relevant pathways including several RTKs, key modulators of the TME interplay, as well as nodal epigenetic and transcriptional regulators 54.

To explore the therapeutic potential of the HS mimetic-based approach in DDLPS, we employed the investigational 2,3-O desulfated non-anticoagulant heparin CX-01 characterized by a multitargeting profile and robust immunomodulatory activity beyond heparanase inhibition 22, 38, 48. Based on these properties, CX-01 has undergone clinical evaluation (Phases 2, 3) in patients with hematological tumors and severe COVID-19 infection 23, 55. Early clinical trials revealed an acceptable safety profile and activity for CX-01 in hematological malignancies 23, 24. Furthermore, in neuroblastoma models, CX-01 exhibited anti-tumor and anti-metastatic activities along with differentiation properties by rewiring pathways involved in tumor progression 56.

Cell surface HSPGs play an accessory role in several ligand-RTK complexes. FGF/FGFR and HGF/c-Met axes are prototypic examples of such cooperation where the interaction with HS chains is mandatory to exert full biological activity 39. Our findings indicate that CX-01 can antagonize the invasive ability of DDLPS cells stimulated by both bFGF and HGF. Moreover, prolonged exposure to this drug reduced the levels of the HSPG SDC1, a result that is consistent with a previous report which showed that SDC1 expression is regulated by the FGF pathway and downregulated upon the induction of differentiation in adipocyte progenitors 13. Our findings, showing a reduction of DDLPS cell growth upon *SDC1* silencing, further support the role of this HSPG as a proliferation promoter, consistent with SDC1 involvement in liposarcomagenesis and progression 13. A protective role against lipid accumulation was previously suggested for c-Met in other pathological conditions such as non-alcoholic steatohepatitis 57. Here, for the first time, we demonstrate that c-Met signaling exerts an anti-adipogenic effect in DDLPS cells.

Both FGFR and c-Met signaling converge downstream upon AKT activation, another nodal point hampering DDLPS cell differentiation and affected by CX-01 treatment. Dysregulation of RTK axes may lead to aberrant activation of AKT in DDLPS 3, 4, 30, 43. The FGFR pathway is frequently deregulated in liposarcomas due to *FRS2* amplification or overexpression/mutation of receptor isoforms 3, 46. Aberrant HGF/c-Met signaling has also been implicated in DDLPS pathobiology through ligand-dependent or -independent mechanisms 30, 43. The central role for oncogenic AKT signaling in the molecular pathogenesis of WDLPS and DDLPS was also demonstrated by Gutierrez and colleagues in a zebrafish liposarcoma model 44.

The heparanase/HSPG axis, including SDC family members, can variably impact the balance between adipocyte hyperplasia and hypertrophy as well as lipid uptake and metabolism 12. Our *in vitro* study supported a direct role of heparanase catalytic activity in the inhibition of lipid production/accumulation contributing to DDLPS cell dedifferentiation. Consistently, a reduction of HS chains has been reported to impair adipocytic differentiation 12. CX-01-induced reactivation of the adipogenic program in DDLPS cells was associated with a reduced histone H4 acetylation, plausibly reflecting a direct inhibition of p300 acetyltransferase and/or an indirect effect through heparanase inhibition 22, 47. These findings are consistent with a positive regulation of adipogenesis promoted by histone deacetylases 41. *In vitro* studies also indicate that CX-01 shares pro-differentiating effects with other non-anticoagulant heparins despite remarkable structural differences. Although these findings suggest a common mechanism of action, deepened SAR studies are needed to delve into structural determinants of anti-DDLPS activities.

Our findings indicate a role for the heparanase/SDC1 system in supporting proliferation and inhibition of differentiation of DDLPS cells. It is noteworthy that several SDC1-targeted therapeutic approaches are currently under clinical investigation in multiple myeloma, a cancer that expresses high levels of the HSPG 58, suggesting that SDC1 may represent a druggable target also in DDLPS. Indeed, the transcriptomic analysis of LS-BZ-1 PDXs provided an *in vivo* validation of the inhibitory effect of CX-01 treatment on *SDC1* and *SDC1*-related molecules/pathways. GSEA also indicated an enrichment of DEGs in the Hallmark gene sets Adipogenesis and Fatty acid metabolism, supporting the heparin derivative pro-differentiation effect pin-pointed in the present study. Furthermore, among the downregulated genes in the Hallmark Inflammatory response gene set, *CXCL8* is transcriptionally regulated by the CXCR4/CXCL12 axis, a known CX-01 target 51. These findings are in accord with the previously described anti-inflammatory effect of CX-01 38, 47, 51. Moreover, there was a significant enrichment of the TGF-β signaling gene set and, consistently, inhibition of TGF-β signaling was confirmed in CX-01-treated cells. Indeed, TGF-β-induced SMAD signaling which negatively regulates adipogenesis during embryonic development 59, plays a role in sustaining proliferation and impairing the adipogenic potential of both adipocyte precursors and DDLPS 5, 11. Furthermore, high levels of TGF-β correlate with the emergence of the DD component of DDLPS from tumor adipocyte stem cells, also contributing to the establishment of a highly immunosuppressive TME 5.

Overall, our findings support the view that, through an HS mimetic mechanism, CX-01, and likely other non-anticoagulant heparin derivatives, can unlock the differentiation program exploiting plasticity features, present in adipocyte stem cells and maintained in DDLPS cells. The concept that drug treatment converted the DDLPS PDX phenotype towards a less aggressive behavior was further confirmed by the substantial downregulation of a 29-gene set reported by Wang *et al*., 36 via the integration of lipidomic and transcriptomic analyses. Genes included in this signature, overexpressed in DDLPS compared to WDLPS clinical samples, were indicated by the authors as potential biomarkers discriminating between the two liposarcoma types. Interestingly, genes in the above 29-list, *TIMP1, FN1, COL5A1,* and* MMP14,* were consistently downregulated after CX-01 treatment of DDLPS PDXs also in other gene sets. *TIMP1* has been described as a negative regulator of adipogenesis in adipose-derived stem cells 60, and its high expression in DDLPS correlated with poor patient survival 61. *FN1* is involved in cell adhesion and migration processes. A FN1-rich matrix inhibited preadipocyte differentiation to mature adipocytes by maintaining cellular adhesion and fibroblastic preadipocyte morphology. *FN1* expression is downregulated during adipocyte differentiation 62 according to our finding in CX-01-treated DDLPS PDX. MMP14 influences cell-cell and cell-ECM interactions mediating processes such as ECM degradation and remodeling, cell invasion, and metastasis also via SDC1 cleavage. It regulates lipid metabolism through modifying the pericellular microenvironment and promoting protein shedding 63, 64. *MMP14* was listed in an 11-gene predictor for distant recurrence free survival in liposarcoma patients 65. *COL5A1* has been recently identified as a risk gene included in a six-gene prognostic signature of liposarcoma generated through machine learning combining DEGs between liposarcoma and normal adipose tissues present in public datasets 66. Notably, we found, in independent clinical cohorts, significantly higher expression levels of *TIMP1, FN1, COL5A1, MMP14* and *SDC1* in the DD component of DDLPS specimens compared to the WD component and normal fat, suggesting an association with disease progression.

In conclusion, the current study demonstrates the potential of a multitargeting approach based on HS competition to simultaneously inhibit multiple antiadipogenic players, prompting an enhanced differentiated phenotype compatible with a less aggressive DDLPS behavior. Mechanistic studies into CX-01 activities implicate regulatory networks with a pathobiological role in DDLPS, representing druggable vulnerabilities. We propose that non-anticoagulant heparins including CX-01, may achieve a substantial contribution in counteracting DDLPS dedifferentiation, thereby diminishing tumor aggressiveness.

## Supplementary Material

Supplementary figures and tables.

## Data Availability

The data generated in this study (i.e. gene expression data of controls and CX-01-treated LS-BZ-1 PDXs), are publicly available at Gene Expression Omnibus (GEO) under accession number GSE307802.

## Abbreviations

COL1A5: collagen type V alpha 1 chain
ECM: extracellular matrix
HS: heparan sulfate
HSPG: heparan sulfate proteoglycan
DDLPS: dedifferentiated liposarcoma
DEG: differentially expressed genes
FABP4: fatty acid-binding protein 4
FC: log_2_ fold change
FDR: false discovery rate
FGF: fibroblast growth factor
FN1: fibronectin 1
FRS2: fibroblast growth factor receptor substrate 2
HGF: hepatocyte growth factor
HAT: histone acetyltransferase
MMP14: matrix metalloproteinase 14
ORO: Oil Red O
PDX: patient-derived xenograft
PPAR-γ: peroxisome proliferator-activated receptor gamma
RTK: receptor tyrosine kinase
SDC1: syndecan 1
SREBP1: sterol-regulatory-element-binding-protein-1
ssLMWH: super-sulfated low molecular weight heparin
TGFβ: transforming growth factor beta
TME: tumor microenvironment
TIMP1: TIMP metallopeptidase inhibitor 1
TV: tumor volume
TVI: tumor volume inhibition
WDLPS: well differentiated liposarcoma
