# Supplementary material for: Targeting the heparan sulfate proteoglycan/heparanase axis as a novel therapeutic strategy to drive adipogenesis and block tumor growth of dedifferentiated liposarcoma
Source: Int J Biol Sci. 2026 Aug 11;22(13):7173–90. doi: 10.7150/ijbs.133802 (PMC13540702; doi:10.7150/ijbs.133802)
Supplement: Supplementary file 1 [file ijbsv22p7173s1.pdf]

SUPPLEMENTARY INFORMATION

Targeting the heparan sulfate proteoglycan/heparanase axis as a novel therapeutic strategy to drive adipogenesis and block tumor growth of dedifferentiated liposarcoma.

Cinzia Lanzi, Enrica Favini, Laura Dal Bo, Monica Tortoreto, Stefano Percio, Valentina Zuco, Gian Paolo Dagrada, Silvia Brich, Paola Collini, Loris De Cecco, Dario Callegaro, Roberta Sanfilippo, Alessandro Gronchi, Nadia Zaffaroni, Yehuda G. Assaraf, Sandro Pasquali*, Giuliana Cassinelli*

SUPPLEMENTARY MATERIALS AND METHODS

In vitro assays

For the anchorage-independent growth assay, cells, seeded at 1000-3000 cells/cm^2, were grown in semi-solid agar in the presence or absence of the drug. After 7-14 days, colonies were stained with vital dye and counted under a magnifying projector as previously described [20].

For the invasion assay, cells, pretreated with the indicated drug in complete medium for 24h, were harvested and transferred to the upper chamber of 24-well Transwell plates (Costa, Corning Inc., Corning, NY, USA) previously coated with Growth Factor Reduced Matrigel (Suppl. Table 1) (6–18 x 10^4 cells/filter, according to the spontaneous invasive ability). The same drug concentration used for cell pretreatment was added to both the upper and lower chambers. Where indicated, human recombinant growth factors were added into the lower chamber at 50 ng/ml (Suppl. Table 1). After 24h, cells that invaded Matrigel were stained with sulforhodamine B and counted under an inverted microscope as described [19]. Alternatively, cells (20000 cells/well) were layered onto Matrigel-coated wells (8.1 µg/ml) in 48-well plates in serum free medium for the branching morphogenesis assay. Photos were taken 8h later by using EVOS XL CORE (Invitrogen, Carlsbad, CA).

For quantification of neutral lipid content, cells, exposed to drugs as indicated, were subjected to BODIPY™ 493/503 staining (Suppl. Table 1) according to the manufacture's instruction. Cells were then harvested and analyzed by flow cytometry using the BD Accuri C6 instrument (Becton, Dickinson, Franklin Lakes, NJ). Data were analyzed using the Kaluza Analysis Software (version 2.1.00002.20011) (Beckman Coulter Life Science, Brea, CA). Alternatively, to avoid interference by small molecule autofluorescence (e.g., SU11274), neutral lipid content was detected by Oil Red O (ORO) staining (Suppl. Table 1). According to the manufacturer's protocol, cells, treated as indicated, were fixed and stained with the dye solution. After cell photo capture by microscope EVOS XL CORE, the dye was dissolved in isopropanol and samples read at 490 nm with iMark microplate reader (Biorad, Hercules, CA). OD readings were corrected for sample protein content (experiments with SU11274, perifosine) or cell number (experiments with OGT2115) measured in parallel.

Biochemical analyses and Western blotting

Proteomic profiling of phosphorylated RTKs in DDLPS cells was performed by using the Human phospho-RTK array kit ARY001B (R&D system, Minneapolis, MN) according to the manufacturer's instructions.

For biochemical analyses, exponentially growing cells were seeded in complete medium and the day after treated with drugs at the indicated concentrations and exposure times. Total cell protein extraction and Western blotting were performed as previously described in detail [20]. Antibodies used are listed in Suppl. Table 1. Otherwise, Western blot analysis was performed on lysates from frozen PDX tumors analogously processed after pulverization by the Mikro-Dismembrator II (B. Brown Biotech International, Melsungen, Germany) [25]. Images from one representative experiment are shown. Loading controls (e.g., vinculin, GADPH, actin) are shown for samples run in different gels. Protein modulations reported were confirmed in at least two independent experiments. Image J 1.46 R (https://imagej. net) was used for the densitometric analysis of blots.

Immunofluorescence microscopy

Cells growing on coverslips were fixed in 2% paraformaldehyde for 15 min and permeabilized in 70% ethanol at -20°C for 20 min. After blocking in 1% BSA in PBS for 1 h, cells were incubated with primary anti-β tubulin (1:500) or -vimentin (1:50) antibody followed by secondary Alexa Fluor 488 or 594 (1:500) anti-mouse antibody. Alternatively, slides were incubated with Fluorescein Isothiocyanate Labeled Phalloidin (1:500) to visualize F-actin. Nuclei were counterstained with Hoechst 33342. Slides, mounted with Prolong Gold Antifade, were examined by a fluorescence microscope equipped with digital camera or the confocal laser scanning microscope Leica TCS SP8 X (Leica Microsystems GmbH, Mannheim, Germany). Suppliers' details for antibodies and reagents are listed in Suppl. Table 1.

Flow cytometry

To assess SDC1 expression levels, cells, harvested at the indicated times, were fixed in 2% paraformaldehyde for 15 min, permeabilized in 70% ethanol at -20°C for 20 min and blocked in 1% BSA in PBS for 1 h. Then, cells were incubated with the primary antibody anti-SDC1(1:50) followed by secondary Alexa Fluor 488 anti-rabbit antibody (1:400). Samples were analyzed using a BD FACSCanto flow cytometer (Becton Dickinson, Mountain View, California) and data elaborated using the Kaluza Analysis Software (version 2.1.00002.20011) (Beckman Coulter Life Science, Brea, CA).

FISH, histological, immunohistological and histochemical analyses

The novel LS-MMC-1 cell line was subjected to cytogenetic and FISH analyses for diagnostic confirmation by a sarcoma-dedicated cytogeneticist. FISH was carried out on interphase nuclei from cultured cells using the the ZytoLight SPEC MDM2/CEN 12 Dual Color Probe (PL18) (ZytoVision GmbH, Bremerhaven, Germany). Slides were scored with a Leica DM 6000B (Wetzlar, Germany) microscope at ×1000 magnification and the appropriate fluorescence filters. The images were captured using Cytovision software (v. 7.0, Leica, Wetzlar, Germany).

For morphological analysis and MDM2 expression evaluation, 4 µm sections of LS-MP-1 PDX formalin-fixed paraffin-embedded (FFPE), were stained with hematoxylin and eosin (H&E) and immunostained with anti-MDM2 antibody (Ab-1, mAb, IF2, Calbiochem, San Diego, CA #OP46, dilution 1:40).

Staining of neutral lipid droplets on ex vivo PDX samples was performed according to ORO manufacturer's protocol on frozen tissue sections selected by sarcoma pathologist. Slides were mounted with an aqueous mounting medium and images captured with automated microscope equipped with Metafer 4 software (Metasystems, Altlussheim, Germany).

Supplementary Table 1

Name	Manufacturer	Catalog. N°
Mouse monoclonal antibodies		
Syndecan (A-6)	Santa Cruz Biotechnology, Santa Cruz, CA	sc-390791
tubulin	Sigma-Aldrich, St. Louis, MO	T-4026
GAPDH	Sigma-Aldrich, St. Louis, MO	G8795
vinculin	Sigma-Aldrich, St. Louis, MO	V-9264
Adiponectin	Abcam, Cambridge, UK	ab22554
MET (25H2)	Cell Signaling Technology, Beverly, MA	3127
AKT	BD Transduction, Lexington, KY	610861
CDK4(DCS-35)	Santa Cruz Biotechnology, Santa Cruz, CA	23896
MDM2 (SMP-14)	Santa Cruz Biotechnology, Santa Cruz, CA	sc-965
Rabbit monoclonal antibodies		
FABP4	Abcam,Cambridge,UK	ab92501
Phospho-MET (Y1234/1235)	Cell Signaling Technology, Beverly, MA	3077
TIMP-1 (D10E6)	Cell Signaling Technology, Beverly, MA	8946
Vimentin (for IF)	Dako, Agilent, Santa Clara, CA	M0725
Rabbit polyclonal antibodies		
actin	Sigma-Aldrich, St. Louis, MO	A 2066
Heparanase 1	Abcam,Cambridge,UK	ab42817
phospho-p44/42MAP Kinase (Thr202/Tyr204)	Cell Signaling Technology, Beverly, MA	9101
p44/42 MAPK	Cell Signaling Technology, Beverly, MA	9102
phospho-Akt (Ser473)	Cell Signaling Technology, Beverly, MA	9271
acetyl histone H4 (Lys12)	Cell Signaling Technology, Beverly, MA	2591
SREBP1	Abcam, Cambridge, UK	ab28481
Phospho-FRS2(Y436)	R&D system, Minneapolis, MN	AF 5126
FRS2(H81)	Santa Cruz Biotechnology, Santa Cruz, CA	sc8318
Phospho-GSK3 α/β (ser21/9)	Cell Signaling Technology, Beverly, MA	9331
GSK3 β	Santa Cruz Biotechnology, Santa Cruz, CA	sc9166
PSMAD2 (S465/467)	Cell Signaling Technology, Beverly, MA	3108
SMAD2	Cell Signaling Technology, Beverly, MA	5339
Fibronectin 1	Abcam, Cambridge, UK	Ab32419
CD138 (Syndecan-1) (FACS)	Invitrogen, Waltham, MA	36-2900

Secondary antibodies	Manufacturer	Catalog. N°.
Anti-mouse IgG HRP-linked	Cell Signaling Technology, Beverly, MA	7076
Anti-rabbit IgG HRP-linked	Cell Signaling Technology, Beverly, MA	7074
Goat anti-Mouse IgG (H+L) Superclonal™ Secondary Antibody, Alexa Fluor™ 488	Invitrogen, Waltham, MA	A28175
Goat anti-Mouse IgG (H+L) Cross-Adsorbed Secondary Antibody, Alexa Fluor™ 594	Invitrogen, Waltham, MA	A11005
Goat anti-Rabbit IgG (H+L) Cross-Adsorbed Secondary Antibody, Alexa Fluor™ 488	Invitrogen, Waltham, MA	A11008

Drugs/Reagents	Manufacturer	Catalog. N°.
SU11274	SelleckChem, Houston, TX	S1080
OGT2115	Santa Cruz Biotechnology, Dallas, TX	sc-204144A
Perifosine	SelleckChem, Houston, TX	S1037
HGF	Sigma, St. Louis, MO	H1404
bFGF	Peprotech, Rocky Hill, NJ	100-18B
BODIPY™ 493/503	Molecular Probes, Eugene, OR	D3922
Oil Red O (ORO)	ScienCell, Carlsbad, CA	0843a
Prolong ™ Gold antifade	Invitrogen, Waltham, MA	P10144
Hoechst 33342	Invitrogen, Waltham, MA	H3570
sulforodhamine B (SRB)	Sigma, St. Louis, MO	S-9012
Growth Factor Reduced Matrigel	BD Biosciences, San Jose, CA	356231

siRNA

Name	Sequence (5'-3')	Manufacturer	Catalog. N°.
siSDC1-1	(sense GGAGGAAUUCUAUGCCUGAtt) (antisense UCAGGCAUAGAAUUCCUCCtg)	Ambion, Austin, TX	12527
siSDC1-2	(sense GCAGGUGCUUUGCAAGAUAtt) (antisense UAUCUUGCAAAGCACCUGCac)	Ambion, Austin, TX	s12634
siSDC1-3	(sense CCAUUCUGACUCGGUUUCUtt) (antisense AGAAACCGAGUCAGAAUGGtg)	Ambion, Austin, TX	142557
Negative control siRNA (Silencer Select #2)		Invitrogen, Waltham, MA	4390846

Assays

Name	Manufacturer	Catalog. N°
GAPDH PrimeTime Std qPCR assay	Integrated DNA Technologies, IDT, Coralville, IA	235728378
SDC1 PrimeTime Std qPCR assay	Integrated DNA Technologies, IDT, Coralville, IA	235728382
HPSE PrimeTime Std qPCR assay	Integrated DNA Technologies, IDT, Coralville, IA	384209788

Supplemental Table 2. Leading Edge Genes of statistically significant GSEA of RNA-seq data comparing whole transcriptomes of tumor samples from LS-BZ-1 PDX control and treated with CX-01.

	LEADING EDGE GENES	Additional DEGs involved in the process/pathway
**HALLMARK GENE SETS**		
**Adipogenesis**	STOM, NDUFS3, NDUFAB1, ARL4A, MTCH2, CMPK1, DNAJC15, PRDX3, DHRS7B, UQCR11, UCP2, CHUK, CD36, CYC1, NDUFB7, SDHC, MGST3, NDUFA5, COQ3, ATP1B3, ATL2, AK2, PPARG, SDHB, IFNGR1, SLC66A3, COX6A1, UQCR10, MRPL15, COX7B, SOD1, SCP2, MTARC2, COX8A, CD302	**SDC1**, **FGF2**, **TIMP1**, **FN1,** MYLK, MYH3, TNFSF12, FABP5
**Fatty acid metabolism**	H2AZ1, CBR1, ODC1, PTS, SDHD, PCBD1, PDHB, ECI2, BPHL, ACOT8, MIX23, CD36, SDHC, MDH1, NTHL1, CBR3, ELOVL5, S100A10, HSDL2, CRYZ, YWHAH, MAOA, HCCS, AUH, ADSL, UROD, UROS, ACAT2, SUCLG1, BLVRA, SUCLA2, ALDH3A2, ENO3, NCAPH2, ACAA1, GLUL RAP1GDS1, GCDH	
**Inflammatory response**	IRAK2, RELA, **MMP14**, ICAM1, NFKBIA, NFKB1, CSF1, CD82, TNFRSF1B, RHOG, EDN1, IRF1, OSMR, RIPK2, ATP2A2, NAMPT, **PLAUR**, SLC11A2, SLC4A4, AHR, LPAR1, TLR3, SLC7A1, **TIMP1**, TAPBP, BEST1, LIF, LDLR, CXCL8, IL1R1, IL4R, PSEN1, ADRM1, ITGA5, MYC, ABI1, TPBG, IRF7, SLC1A2, HBEGF, SELENOS, DCBLD2	CXCL1, PTGS2, NFKB2, C1QTNF1, NFKBIZ, IL32, TNFSF4, RELB, BCL3
**TGF β signaling**	NCOR2, LTBP2, HIPK2, TJP1, SMAD7, SPTBN1, WWTR1, SMURF1, ENG, JUNB, SMAD6, SMURF2, BMPR2, CDK9, ID3, ARID4B, SKI, SKIL, SLC20A1, PPP1R15A, XIAP, SERPINE1	TGFBR2, LRRC32, **MMP14**, **TIMP1**
**OTHER GENE SETS**		
**WANG et. al. [36]**<br>**Secreted protein coding genes**	**FN1,** EMILIN1, THBS2, FBN1, **PLAUR,** TRIO, **COL5A1, TIMP1,** ECM1, P4HA2**. MMP14**	
**PID**<br>**syndecan-1 pathway**	BSG, CASK, CCL5, COL10A1, COL11A1, COL11A2, COL12A1, COL13A1, COL14A1, COL15A1, COL16A1, COL17A1**, COL1A1**, **COL1A2**, COL2A1, **COL3A1**, COL4A1, COL4A3, COL4A4, COL4A, COL4A6, **COL5A1**, **COL5A2**, COL6A1, COL6A2, COL6A3, COL7A1, COL8A1, COL8A2, COL9A1, COL9A2, COL9A3, HGF, HPSE, LAMA5, MAPK1, MAPK3, MET, MMP1, MMP7, MMP9, PPIB, PRKACA**, SDC1**, SDCBP, **TGFB1**	
**REACTOME**<br>**syndecan-1 interactions**	ACTN1, CASK, **COL1A1**, **COL1A2, COL3A1**, **COL5A1**, **COL5A2**, COL5A3, **FGF2**, **FN1**, ITGA2, ITGA6, ITGAV, ITGB1, ITGB3, ITGB4, ITGB5, PRKCA, **SDC1**, SDC2, SDC3, SDC4, **TGFB1**, THBS1, TNC, TRAPPC4, VTN	

Blue, genes downregulated by treatment; **red**, genes upregulated by treatment; **bold**, genes shared in multiple gene sets

Supplementary Figure 1

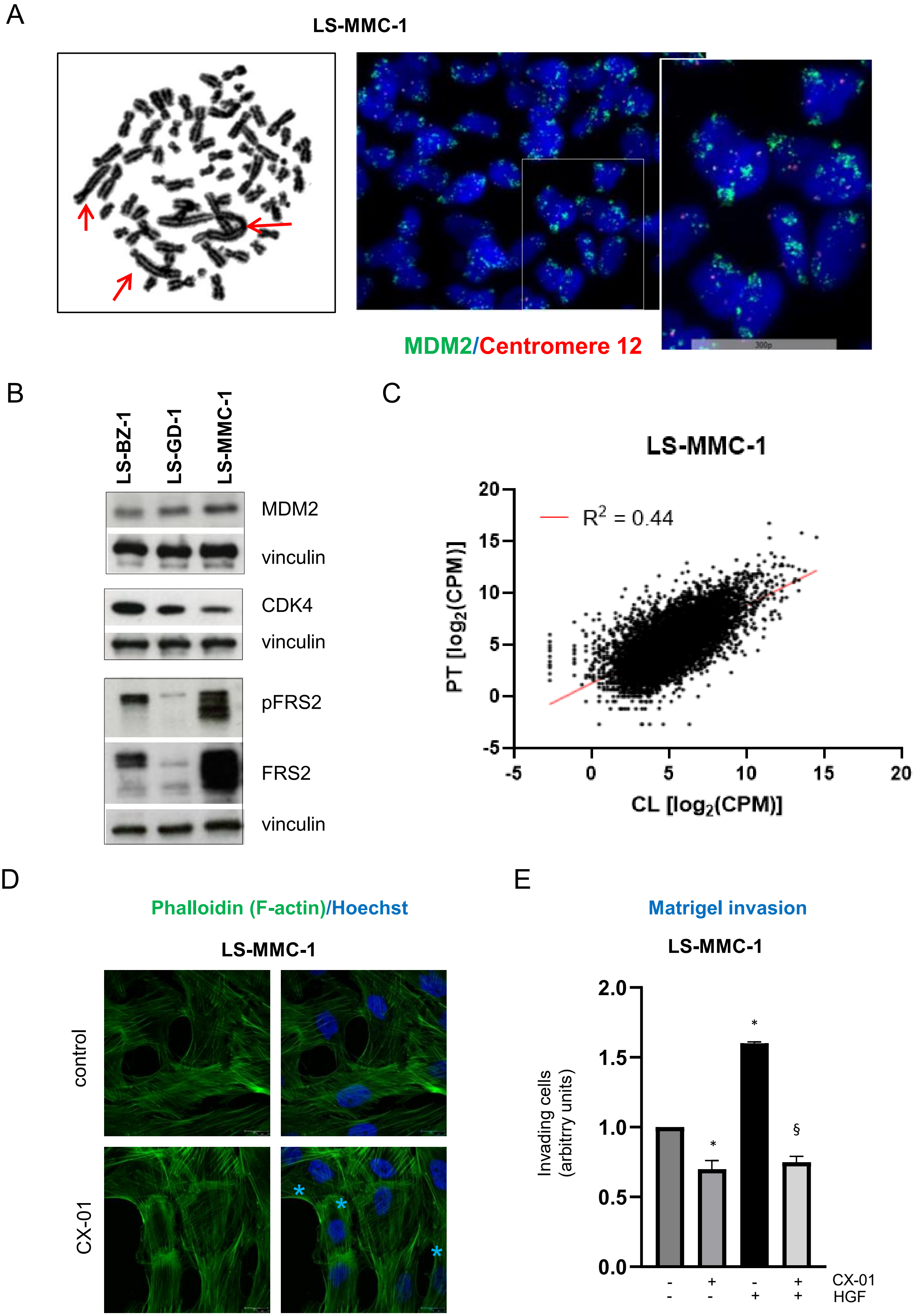

Suppl. Fig. 1. Characterization of the novel DDLPS cell line LS-MMC-1 and response to CX-01 treatment. A) Left panel, G banded metaphase spread from cultured LS-MMC-1 cells showing hyperdiploidy and giant chromosomes (red arrows). Right panel and inset, interphase nuclei from LS-MMC-1 cells hybridized with MDM2 (Spectrum Green) and centromere 12 (Spectrum Orange) probes showing large clusters of amplified MDM2 gene copies. DAPI counterstaining highlights nuclei. B) Western blots showing MDM2, CDK4 and FRS2 expression levels along with FRS2 phosphorylation in DDLPS cell lines. Anti-vinculin blots are shown as loading controls. C) Scatter plot showing correspondence of the RNA-Seq transcriptomic profiles between the patient's primary tumor (PT) and the LS-MMC-1 cell line (CL). Linear model trend (red line) and Spearman'correlation coefficient ($r_s = 0.65$, $P<0.0001$) were calculated.

D) Filamentous actin (F-actin) was stained in control and CX-01-treated LS-MMC-1 cells (1 mg/ml, 72 h) by using phalloidin (green). Asterisks indicate cage-like structures accommodating lipid droplets. Nuclei are evidenced by Hoechst 33342 counterstaining (blue). Representative images captured with a confocal fluorescent microscopy are shown (original magnification 63x).

E) After pretreatment with 1 mg/ml CX-01 for 24h, LS-MMC-1 cells were transferred onto Matrigel-coated Transwell chambers and incubated for an additional 24h with (+) or without (−) HGF (50 ng/ml). Invading cells are reported as arbitrary units ± SD from three independent experiments. *, $p < 0.05$ vs serum starved control; §, $p < 0.05$ versus HGF-stimulated cells.

Supplementary Figure 2

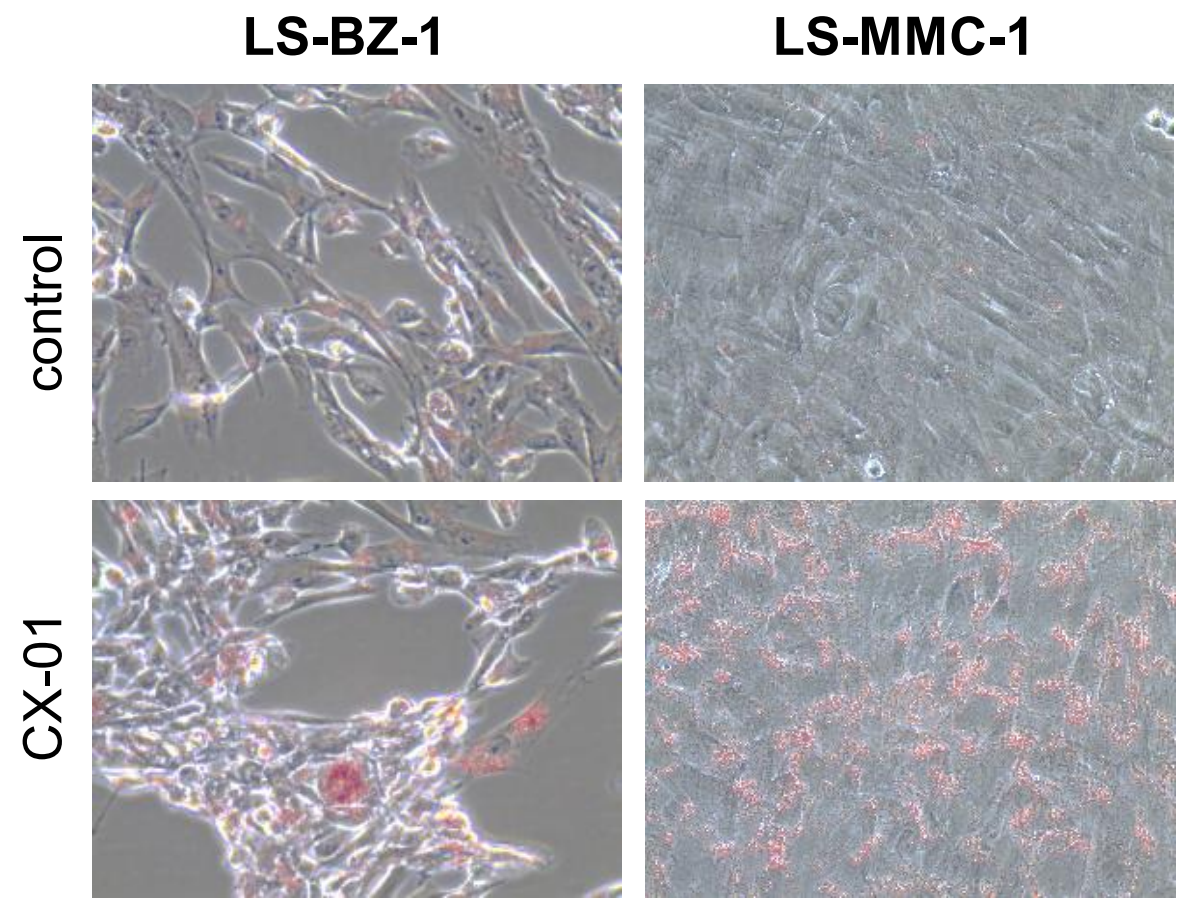

Suppl. Fig. 2. CX-01 enhances lipid content in DDLPS cells. A) Representative images of ORO stained control and CX-01-treated cells (1 mg/ml). LS-BZ-1 cells were exposed to the heparin derivative for 72h. LS-MMC-1 were exposed to CX-01 for 72h then medium was replaced and drug treatment repeated for additional 96h (original magnification 40x).

Supplementary Figure 3

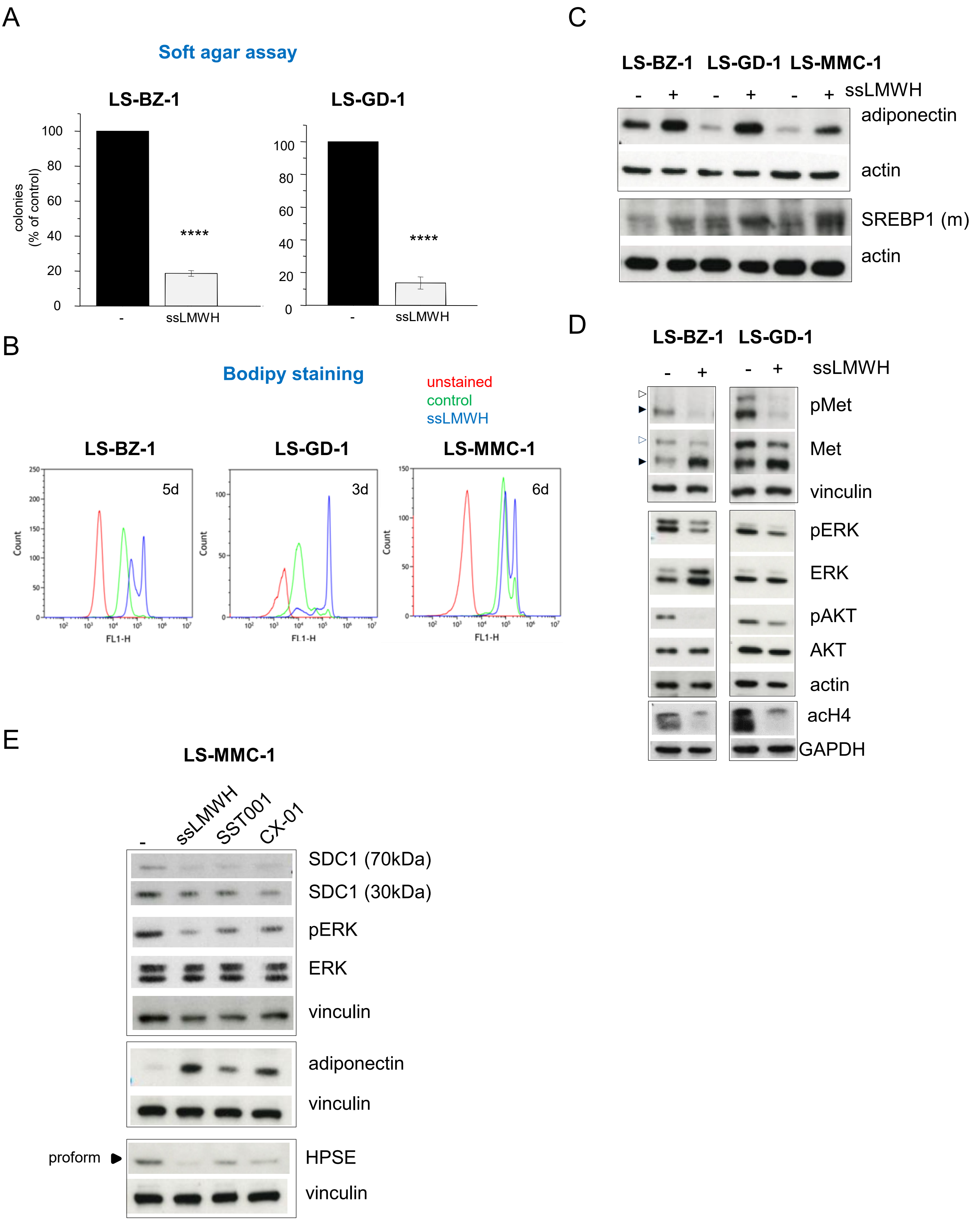

Suppl. Fig. 3 Super sulfated low molecular weight heparin (ssLMWH) inhibits DDLPS cell anchorage-independent growth and promotes adipocytic differentiation. A) Soft agar assay. Cells were seeded in soft agar and incubated in the presence or absence of 1 mg/ml ssLMWH for 7-10 days, Colonies were then stained with a vital dye and counted under a magnifying projector. Average data ± SE from three independent experiments, performed in duplicate, are shown. ****, $P < 0.0001$, drug-treated *vs* untreated cells. B) Lipid staining. Control and 0.5 mg/ml ssLMWH-treated cells were collected at the indicated times and incubated with BODIPY 493/503 for lipid content quantification by FACS. Histograms show an increased accumulation of neutral lipids in drug-treated cells. C) , D) Adipogenic marker induction and c-Met signaling inhibition. Cells were exposed to 1 mg/ml ssLMWH for 48h, then lysed and processed for Western blot analysis with the indicated antibodies. C), D) Blots show induction of adiponectin and mature SREBP1(m) as well as inhibition of Met-ERK-AKT pathway and acetylation of histone H4 (K27) in drug-treated cells.

E) Adiponectin induction in parallel with ERK signaling inhibition and downmodulation of SDC1 and heparanase by three structurally different non-anticoagulant heparin derivatives ssLMWHs, SST0001 and CX-01 in LS-MMC-1 cells. After treatment with the drugs (1 mg/ml) for 48h, cells were lysed and processed for western blot analysis with the indicated antibodies. The band of SDC1 migrating at about 70kDa represents the SDS-resistant dimer while the band detected at about 30kDa represents the core protein.

Supplementary Figure 4

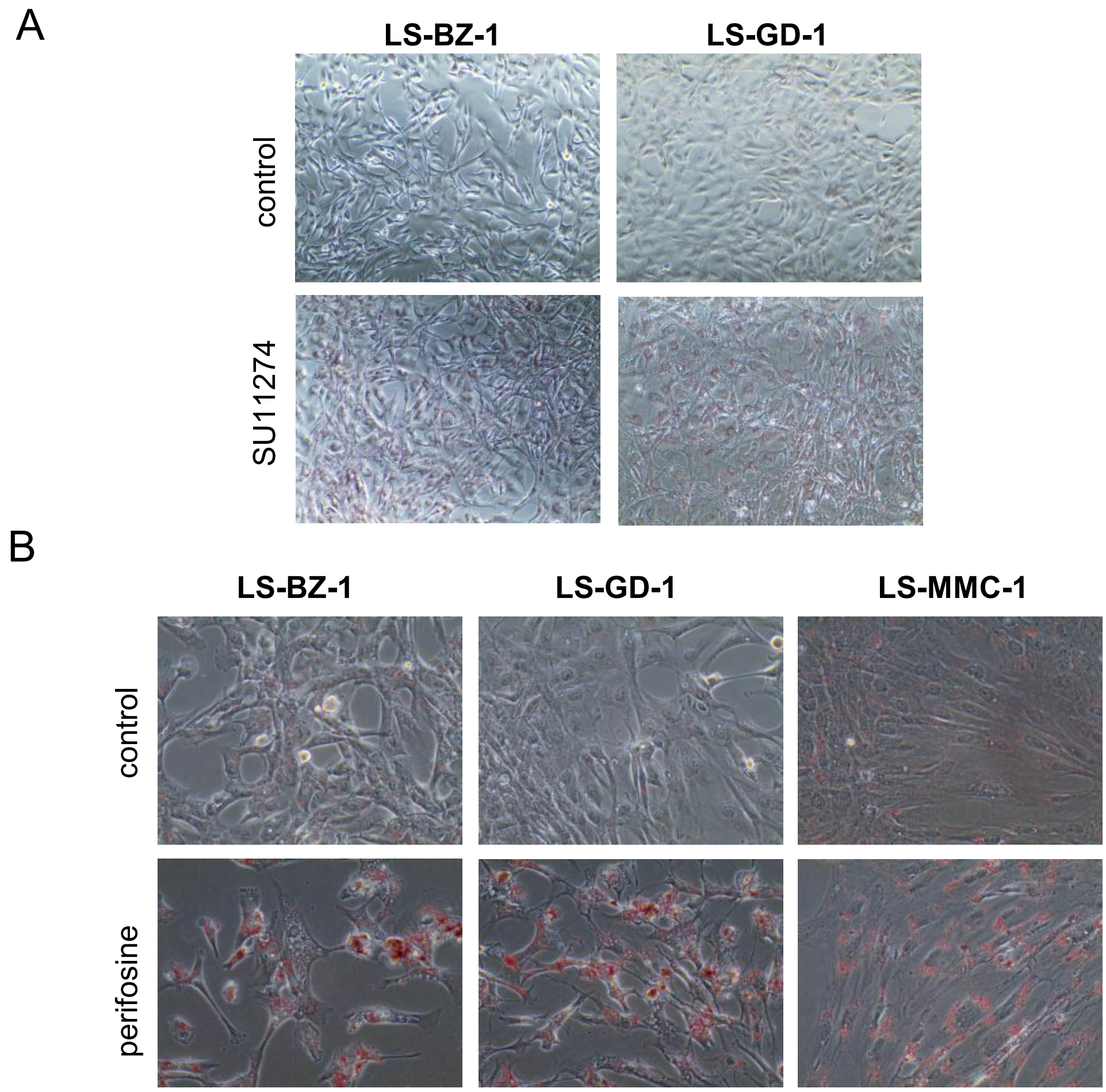

Suppl. Fig. 4 The-Met kinase inhibitor SU11274 and the AKT inhibitor perifosine enhance lipid content in DDLPS cells. A) Representative images of Oil Red O (ORO) stained cells exposed to solvent (control) or 10 uM SU11274 for 24h (original magnification 20x). B) Representative images of ORO stained cells exposed to solvent (control) or 20 uM perifosine for 72h (original magnification 40x).

Supplementary Figure 5

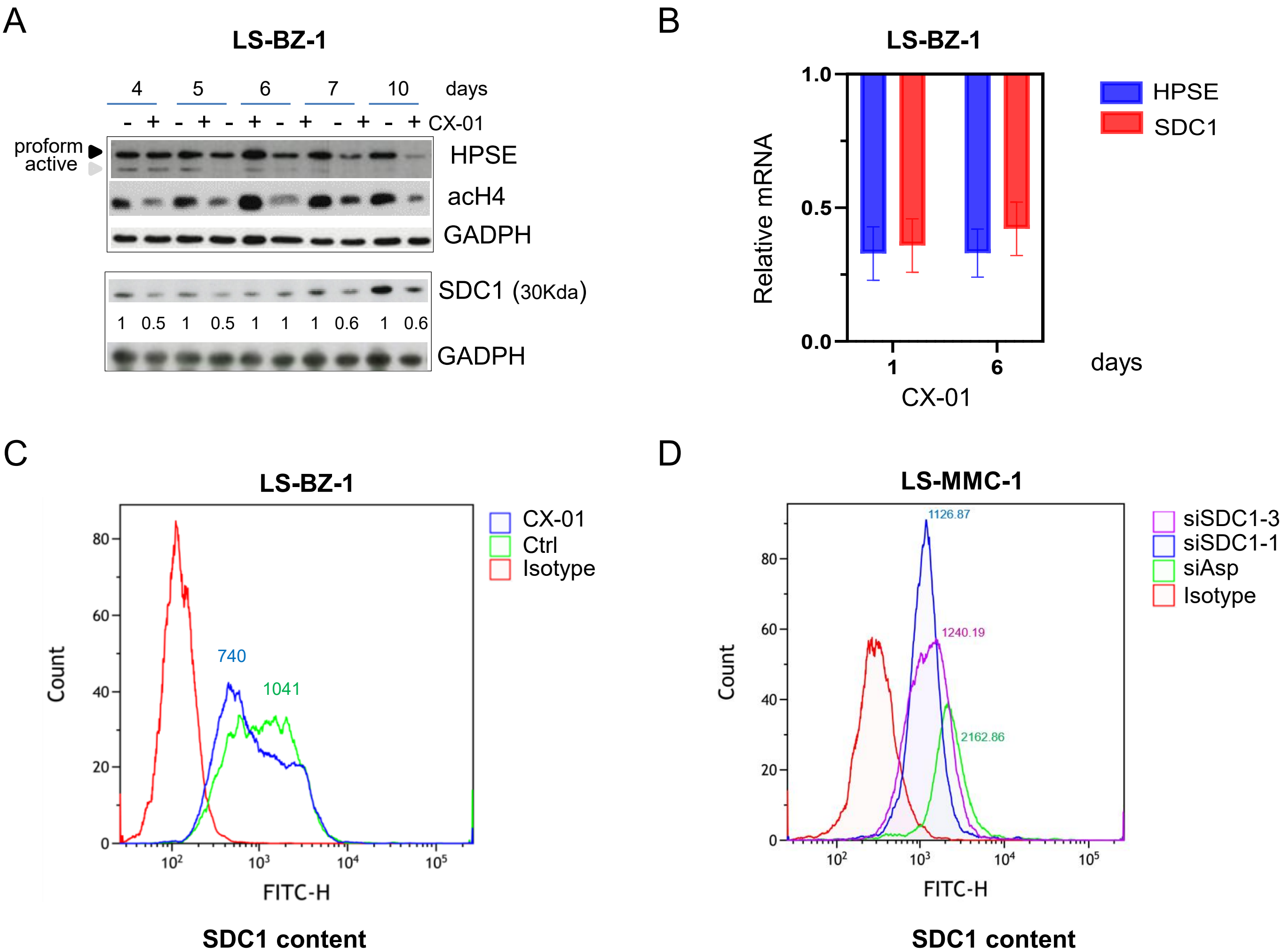

Suppl. Fig. 5. CX-01-induced adipocytic differentiation is associated with heparanase and SDC-1 downregulation. A) LS-BZ-1 cells were first exposed to 1 mg/ml CX-01 for 72h. After medium removal drug treatment was repeated. Western blots show CX-01-induced modulation over time since the first treatment of heparinase (proform and active form) and SDC1 core protein (30kDa). Acetylation of histone H4 (K27) is reported as a marker of HAT inhibition by the heparin derivative. Numbers represent the intensity of relevant bands normalized to the respective loading controls (GADPH blots). B) Analysis of *HPSE* and *SDC1* expression by qRT-PCR in LS-BZ-1 cells treated CX-01 for the indicated times. Data are reported as mean relative quantification ± SD with respect to untreated cells from three independent experiments. C) Reduced content of SDC1 in LS-BZ-1 cells were exposed to CX-01 with respect to untreated cells. LS-BZ-1 cells exposed to CX-01 (1 mg/ml) for 72h, then medium was removed and drug treatment repeated for additional 72h . Collected cells were processed for FACS analysis. D) Reduced expression of SDC1 in LS-MMC-1 cells after transient knockdown of the HSPG by siRNA interference. FACS analysis of SDC1 content was assessed in LS-MMC-1 cells after 72h-transfection with 20 nM non-specific RNA oligonucleotide (siAsp) or SDC1 siRNAs (siSDC1-1 and siSDC1-3. In C) and D), numbers represent Median Fluorescent Intensity within respective cell population.

Supplementary Figure 6

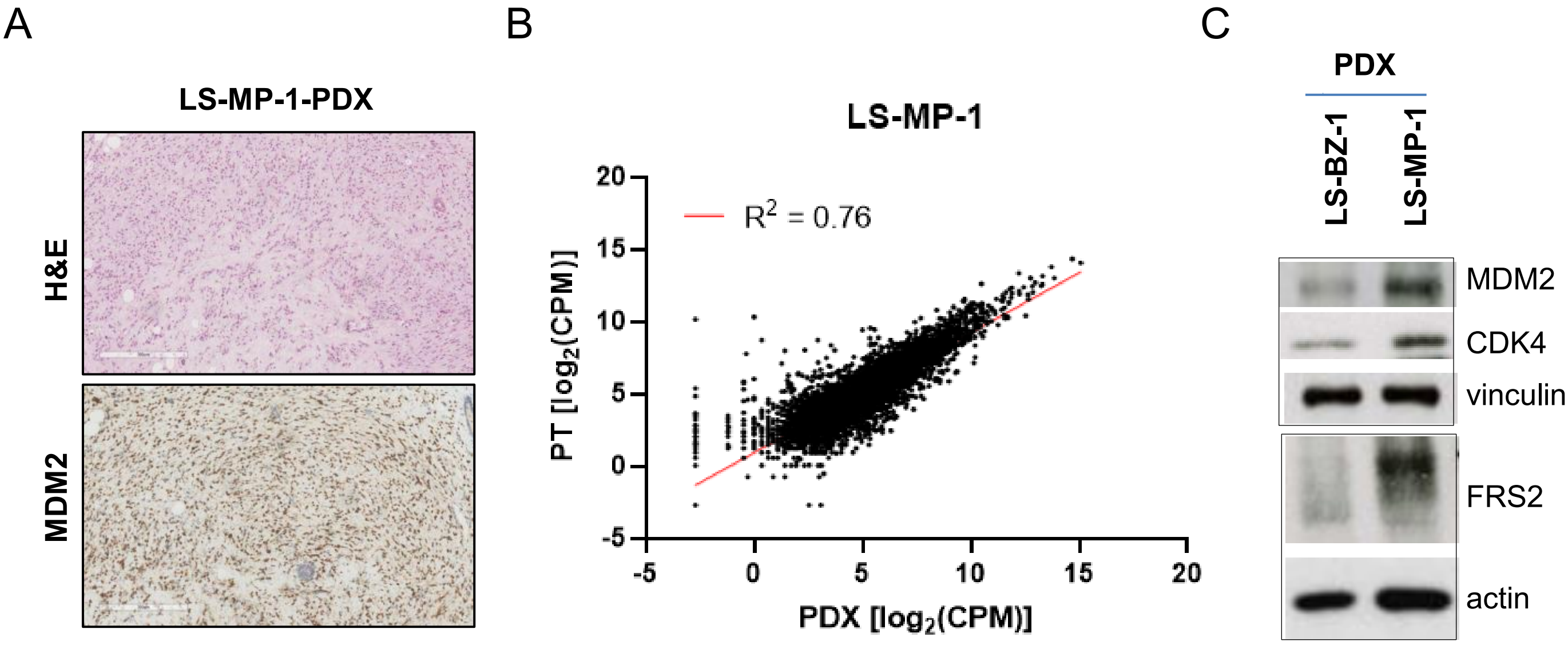

Suppl. Fig. 6 Characterization of the novel DDLPS PDX model LS-MP-1. A) Representative pictures showing LS-MP-1 PDX histology revealed by Hematoxylin and Eosin (H&E) staining, and MDM2 protein detected by immunohistochemistry. B) Scatter plot showing correspondence of the RNA-Seq transcriptomic profiles between the patient's primary tumor (PT) and the LS-MP-1 PDX. Linear model trend (red line) and Spearman'correlation coefficient (r_s = 0.88, P<0.0001) were calculated. C) Western blots showing MDM2, CDK4, FRS2 protein levels in LS-BZ-1 and LS-MP-1 PDXs. Vinculin and actin blots are shown as loading controls.

Supplementary Figure 7

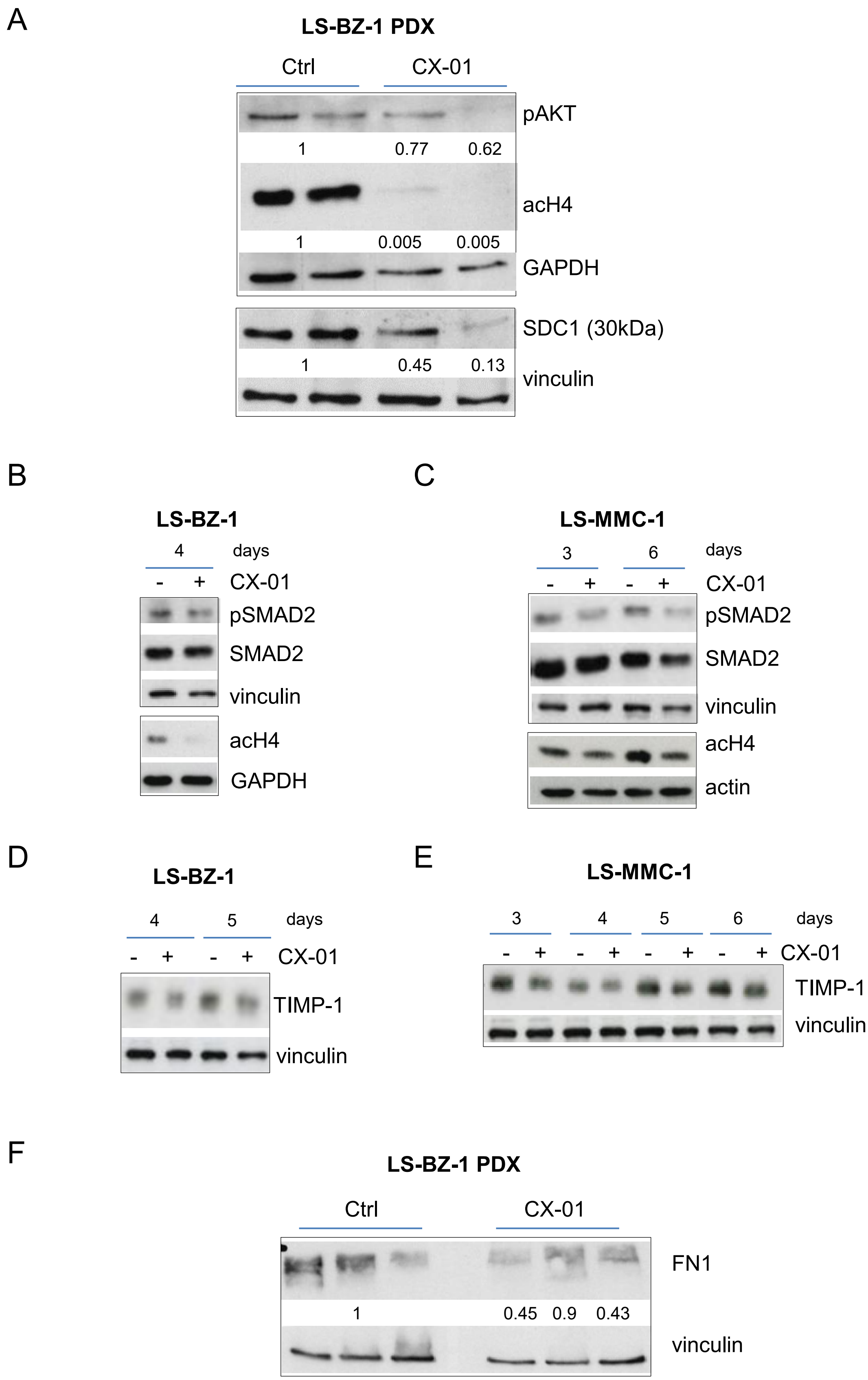

Suppl. Fig. 7. *In vivo* and *in vitro* validation of CX-01-induced modulation of players involved in DDLPS growth and aberrant differentiation.

In A) and F), LS-BZ-1 PDX-bearing mice were treated with s.c. vehicle or CX-01 (30 mg/kg, 2qdx5/w. At the end of experiment, tumors were removed and processed for western blot analysis. A) Inhibition of AKT phosphorylation and H4 acetylation (K27) and downmodulation of SDC1 core protein in CX-01 treated LS-BZ-1 PDX.

B)-E) *In vitro* validation of TGFβ signaling inhibition by CX-01 in DDLPS cells. Drug treatment inhibits phosphorylation of the TGFβ effector SMAD2 (B, C) and reduces levels of the transcriptional target TIMP-1 (D, E). LS-BZ-1 and LS-MMC-1 cells were exposed to 1 mg/ml CX-01 for 72h, then medium was replaced and drug treatment repeated. Days from the first treatment are indicated. In D) and E), acetylation of histone H4 (K27) is shown as marker of HAT inhibition by CX-01.

F) Fibronectin (FN1) downmodulation in CX-01-treated LS-BZ-1 PDX. Numbers represent the intensity of relevant bands normalized to the average intensity of loading controls (vinculin).

Supplementary Figure 8

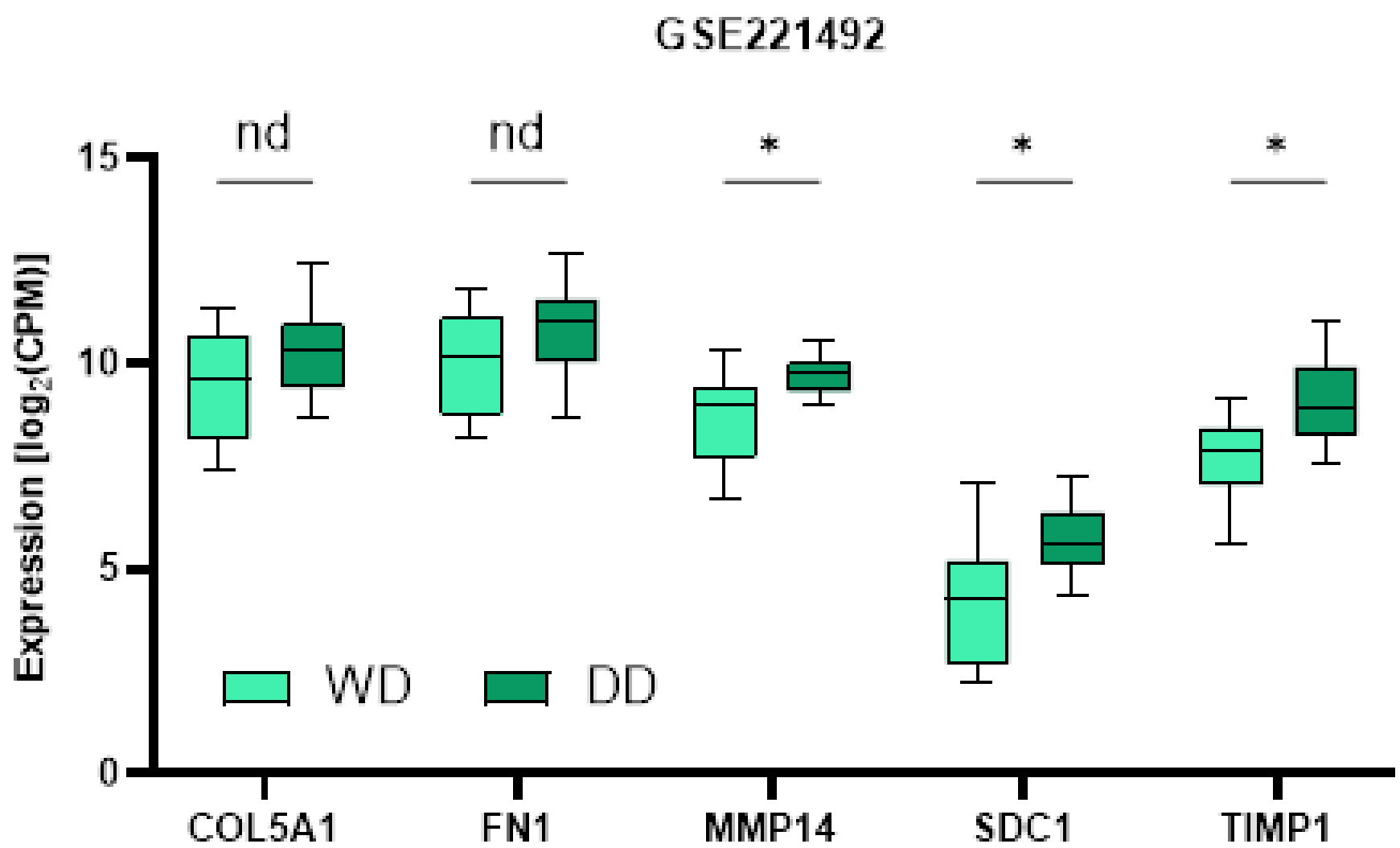

Suppl. Fig. 8 Analysis of RNA-Seq data from retroperitoneal DDLPSs in the independent dataset GSE221492 (n = 15) [5]. Samples of WD and DD tumor components were obtained from the same sites of the tumors. Paired normal fat tissues were not available. Boxplots show an increasing expression of selected genes (COL5A1, FN1, MM14, SDC1 and TIMP1) of the transcriptomic profiles from WD to DD tumor components.
